# Aqueous multiphoton lithography with multifunctional silk-centred bio-resists

**DOI:** 10.1038/ncomms9612

**Published:** 2015-10-16

**Authors:** Yun-Lu Sun, Qi Li, Si-Ming Sun, Jing-Chun Huang, Bo-Yuan Zheng, Qi-Dai Chen, Zheng-Zhong Shao, Hong-Bo Sun

**Affiliations:** 1 State Key Laboratory on Integrated Optoelectronics, College of Electronic Science and Engineering, Jilin University, 2699 Qianjin Street, Changchun 130012, China; 2 State Key Laboratory of Molecular Engineering of Polymers, Department of Macromolecular Science and Laboratory of Advanced Materials, Fudan University, Shanghai 200433, China; 3 College of Physics, Jilin University, 119 Jiefang Road, Changchun 130023, China

## Abstract

Silk and silk fibroin, the biomaterial from nature, nowadays are being widely utilized in many cutting-edge micro/nanodevices/systems via advanced micro/nanofabrication techniques. Herein, for the first time to our knowledge, we report aqueous multiphoton lithography of diversiform-regenerated-silk-fibroin-centric inks using noncontact and maskless femtosecond laser direct writing (FsLDW). Initially, silk fibroin was FsLDW-crosslinked into arbitrary two/three-dimensional micro/nanostructures with good elastic properties merely using proper photosensitizers. More interestingly, silk/metal composite micro/nanodevices with multidimension-controllable metal content can be FsLDW-customized through laser-induced simultaneous fibroin oxidation/crosslinking and metal photoreduction using the simplest silk/Ag^+^ or silk/[AuCl_4_]^−^ aqueous resists. Noticeably, during FsLDW, fibroin functions as biological reductant and matrix, while metal ions act as the oxidant. A FsLDW-fabricated prototyping silk/Ag microelectrode exhibited 10^4^-Ω^−1 ^m^−1^-scale adjustable electric conductivity. This work not only provides a powerful development to silk micro/nanoprocessing techniques but also creates a novel way to fabricate multifunctional metal/biomacromolecule complex micro/nanodevices for applications such as micro/nanoscale mechanical and electrical bioengineering and biosystems.

## Abstract (web summary)

Scientists are increasingly realising the potential for natural materials in micro- and nanofabrication. Here, the authors employ silk-based resists for aqueous multiphoton lithography towards generating intricate structures by femtosecond direct writing.

## Graphical abstract

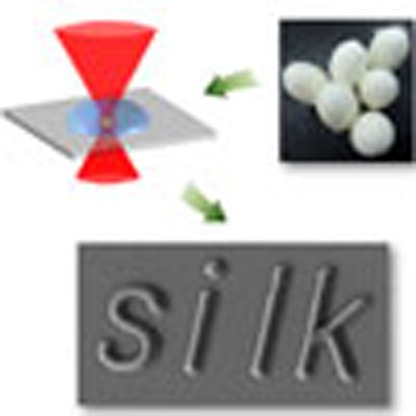

In recent years, the integration of micro/nanoscale processing with natrual biomaterials (for example, proteins^1^,^2^,^3^, peptides^4^ and DNA^5^) has been attracting more and more interest. This booming prospect not only arises from their intrinsic advantages (such as low cost, renewability, biodegradability and eco/biocompatibility)^1^,^2^,^3^,^4^,^5^ but also benifits from the successful combination with other advanced materials during the fabrication process that renders us novel multifunctional hybrid systems for various applications^1^,^2^,^6^,^7^,^8^. Although significant breakthoughs have been achieved recently, some critical research is still needed to be conducted in this area, for instance, mature micro/nanoresolution three-dimensional (3D) fabrication, high mechanical performances of biomacromolecule-based micro/nanostructures and active involvement during ‘top-down' processes not merely as negative matrices. Particularly, organic micro/nanointegration of natural products with other materials such as metals^9^,^10^ is undoubtedly an effective way to realize multifunctional hybrid micro/nanosystems with valuable features (for example, bio/eco-compatibility of both micro/nanoproccessing environments and obtained hybrid systems and flexible customizability of composition, structure and functions)^2^,^4^,^6^,^7^,^8^,^9^,^10^.

As an ancient and FDA-approved natural-product biomaterial, silk and silk fibroin (SF) have been successfully introduced in diverse cutting-edge fields from the well-explored biomedical applications^1^,^2^,^11^,^12^, moving ahead into new-type interdisciplinary miniaturized and integrated biorelated engineering systems (for example, implantable bioelectronics^9^,^10^, optofluidics^13^, organic light-emitting transistors^14^ and micro/nanophotonics^2^,^6^,^7^). Indeed, besides the common advantages of natural biomaterials, SF possesses special additional merits such as remarkable mechanical characteristics, satisfactory optical properties and facile chemical functionalization^1^,^2^. As important cornerstones of burgeoning innovative utilizations of silk, numerous advanced micro/nanofabrication techniques have been tried, such as electron-beam lithography^6^, nanoimprinting^7^, oxygen-based reactive ion etching^15^, ultraviolet lithography^16^, atomic force microscopy (AFM) tip-induced micropatterning^17^, 3D additive printing^18^ and laser micro-ablation^19^. However, two key issues that could greatly limit the further micro/nano-integrated application of SF-based materials still poorly implemented are 3D fabrication with submicroscale or even nanoscale resolution^6^,^7^, and full participation and active functioning in micro/nanostructuring besides merely as device substrate^5^,^6^ and blending matrix^1^,^6^,^7^,^13^. Nevertheless, such problems might be well solved by the assistance of multiphoton lithography technique, namely, femtosecond laser direct writing (FsLDW) via two-photon-adsorption polymerization^20^,^21^,^22^. In addition, the 3D FsLDW approach is endowed with low collateral damage^8^,^20^,^21^, noncontact and maskless capability^23^,^24^, excellent biocompatibility of fabrication process^3^,^8^,^23^,^24^ and versatile applicability for various materials^8^,^21^,^25^,^26^. On the other hand, previous FsLDW-fabricable proteins are comparatively ‘soft'^27^; therefore, SF with higher Young's moduli might bring significant help to adjust mechanical properties of FsLDW-realized, protein-based composite especially for bioapplications such as bone cell culturing and tissue engineering^1^. Moreover, more work is still needed to find convenient method to take full advantage of various functional groups in SF biomacromolecules^28^ and to fabricate SF-based multifunctional hybrid mico/nanosystems for wide applications, such as silk/metals' micro/nanocomposite for bioelectronics. However, until now, to our knowledge, silk-based multiphoton lithography has not been reported and it is still unexplored in an embryonic-like state.

Herein, versatile noncontact and maskless aqueous multiphoton lithography by FsLDW was well realized on ‘configurable' and ‘modularized' diversiform regenerated SF (RSF)-centric inks for different functions (namely, the ink compositions can be designed and adjusted for different functionalities and applications by simply changing or replacing corresponding solution components). First, RSF-based arbitrary 2D (two-dimensional)/3D micro/nanostructures were facilely fabricated, displaying remarkable elastic properties (∼2.2 or 0.22 GPa of the Young's modulus in air or in water, respectively) as well as fine morphology quality (average roughness ∼20–60 nm). Further, ingenious functional silk/metal composite micro/nanodevices can also be skillfully fabricated by FsLDW. The metal content ratio in fabricated silk/metal composite micro/nanostructures could be well controlled by changing metallic ion concentrations^26^, adjusting pH of RSF/[AuCl_4_]^−^ ink^29^ or pre-loading silver (Ag) nanoseeds on fibroin molecules^30^. Consequently, we obtained silk/Ag composite microwires with facilely adjustable electric conductivity. Our work successfully achieved ‘multiple arbitrary customizations' of silk-based micro/nanodevices (composition, geometries, resulted features and functions) even in bioenvironments with live bacteria or cells exsiting nearby, holding great promise to be used in many frontier areas such as bioengineering and bioelectronics.

## Results

### 2D/3D aqueous FsLDW of RSF-based micro/nanostructures

**Figure 1:**
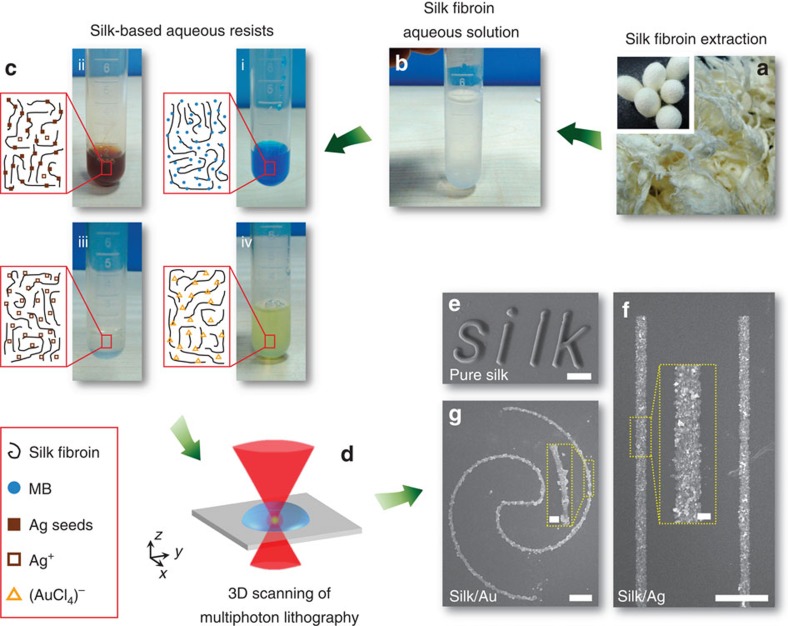
Schematic of FsLDW multiphoton lithography using diversiform silk-based aqueous inks. (**a**) The image of SF extraction from *Bombyx mori* silkworm cocoons in the inset. (**b**) The image of RSF aqueous mother solution (about 3 wt%). (**c**) Diversiform silk-based aqueous inks. (i) RSF/MB aqueous solution; (ii) RSF/Ag nanoseed aqueous solution; (iii) RSF/AgNO_3_ aqueous solution; (iv) RSF/HAuCl_4_ aqueous solution. (**d**) Schematic of 3D scanning of FsLDW multiphoton lithography. (**e**) SEM image of a microscale word of ‘silk' written with RSF/MB aqueous ink; scale bar, 10 μm. (**f**) SEM image of silk/Ag composite microwires fabricated from RSF/AgNO_3_ aqueous ink; scale bar, 10 μm. Inset, enlarged view image; scale bar, 1 μm. (**g**) SEM image of silk/Au composite microwires fabricated with RSF/HAuCl_4_ aqueous ink; scale bar, 10 μm. Inset, enlarged view image; scale bar, 2 μm.

As illustrated in Fig. 1, RSF with the molecular weight ∼50–100 kDa was extracted from *Bombyx mori* silkworm cocoons and then was prepared into aqueous solution with the concentration of 3–5 wt% (see Methods)^31^. After mixing with methlene blue (MB) as a photosensitizer, the all-SF-based aqueous ink (RSF, ∼2.5 wt%; MB, ∼0.017 wt%; pH=7.0) was obtained. Crosslinking of RSF occured with the help of photosensitizer MB in the focal area, where light power intensity is high enough for significant nonlinear optical processes^21^. The probable photochemical mechanism here for SF should be similar to reported multiphoton lithography of oxidizable proteins (bovine serum albumin^3^,^8^,^24^,^27^, avidin^8^,^27^ and lysozyme^27^), which we will disscuss in detail at the end of the manuscript. Finally, by 3D scanning of focused laser spot, RSF-based micro/nanostructures were FsLDW-customized on the substrate (glass slices here) after developing with pure water (Fig. 1e–g). In addition, it was found in our experimnts that FsLDW-fabricated RSF-based micro/nanostructures could not be completely dissolved in 9.5-mol l^−1^ LiBr solution, indicating that FsLDW processing probably induced covalent crosslinking of RSF^31^.

**Figure 2:**
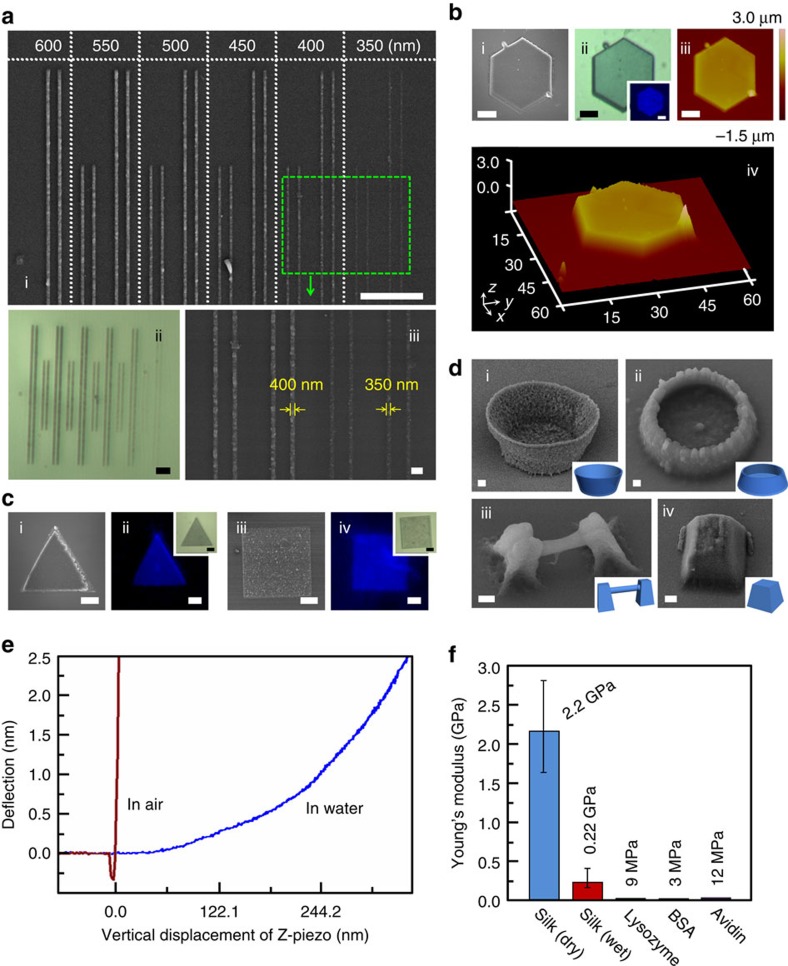
All-silk-based micro/nanostructures customized by FsLDW multiphoton lithography. (**a**) (i) SEM image of parallel all-silk-based single nanowires with line width from ∼350 to ∼600 nm; scale bar, 10 μm. (ii) OM image of all-silk-based single nanowires in 1; scale bar, 10 μm. (iii) Enlarged-view SEM image of all-silk-based single nanowires with line width of ∼350 and 400 nm; scale bar, 1 μm. (**b**) An all-silk-based microhexagon. (i) SEM image; scale bar, 10 μm. (ii) OM image; scale bar, 10 μm; inset, 405-nm-excited fluorescent OM image; scale bar, 10 μm. (iii) AFM graph; scale bar, 10 μm. (iv) 3D-view AFM graph; unit of coordinates, μm. (**c**) All-silk-based microtriangle and microsquare. (i,iii) SEM images; scale bars, 10 μm. (ii,iv) 405-nm-excited fluorescent images; insets. OM images; scale bars, 10 μm. (**d**) SEM images of all-silk-based 3D micro/nanosculptures. (i) A 60° tilted true-3D microbowl; scale bar, 1 μm. (ii) A different type 45° tilted true-3D microbowl; scale bar, 1 μm. (iii) A 45° tilted impending microwire; scale bar, 1 μm. (iv) A 45° tilted microscale frustum of a pyramid; scale bar, 1 μm. (**e**) Deflection–displacement loading force curves of an all-silk-based microsquare dried in air and equilibrated in water. (**f**) Comparison of Young's moduli of various FsLDW-fabricated protein-based biopolymers^27^.

FsLDW parameters were optimized for RSF/MB aqueous resist. Here relatively low contents of RSF (2.5 wt%) and MB (0.017 wt%) were adopted to avoid rapid gelation during laser processing because of RSF's self-gelation feature^1^,^2^ (see Methods and Supplementary Methods). Optimal FsLDW-processing parameters of laser power intensity, scanning step and exposure time on single point were 75.0 mW μm^−2^, 100 nm and 1,000 μs, respectively, in comprehensive consideration of fabrication quality and elapsed time (Supplementary Fig. 1). In Fig. 2a, a set of parallel RSF-based nanowires were computer-aided, designed and FsLDW-fabricated with different line widths (from 350 to 600 nm). Subwavelength nanoscale processing resolution and arbitrary designability have been well achieved. As demonstrated using scanning electron microscopy (SEM, Fig. 2bi), optical microscopy (OM, Fig. 2bii) and AFM (Fig. 2biii,iv), an all-silk 2D regular microhexagon was constructed as designed (thickness, 1.5 μm; roughness average, 14 nm; see Supplementary Fig. 2). In addition, various geometries, for example, triangle and square, respectively, in Fig. 2c, can also be easily customized by silk-based multiphoton lithography (see Fig. 1e, Fig. 2 and Supplementary Fig. 3). Roughness average of as-formed RSF microstructures fluctuated from ∼20 to 60 nm (Supplementary Fig. 3). This might be caused by inherent feature of fibroin that it is easy to agglomerate via regional β-sheet crystallization under disturbances of chemical processing, irradiation, heating and shear force^1^,^2^,^6^. Compared with ultraviolet lithography of silk^16^, it should be noted that the RSF was directly used as completely ‘natural' bioprepolymers without any chemical modification. Interestingly, all FsLDW-fabricated RSF microstructures exhibited photoluminescence feature different from original RSF and raw silk. For example, the RSF microstructures emitted blue fluorescence under 405-nm excitation, as shown in Fig. 2bii,cii,civ.

Importantly, with multiphoton lithography, RSF-based 3D microstructures were obtained facilely using FsLDW (Fig. 2d). Specifically, two kinds of true-3D bowls, a suspended microwire with a diameter of 760 nm and the microfrustum of a pyramid were FsLDW-fabricated from RSF-based aqueous ink as exhibited in Fig. 2di–iv, respectively (for structure details see Methods). This distinctive capability of true-3D micro/nanofabrication with RSF owes to inherent features of FsLDW multiphoton lithography. Such a breakthrough might offer great convenience and opportunities to bring silk-based applications in, for instance, micro/nanoscale integrated biosystems, micro-niche cell culturing, and cell guidance and patterning into 3D era.

### Elastic properties of FsLDW-fabricated RSF microstructures

Using AFM indentation^32^, RSF-based microstructure that was FsLDW-fabricated here was proved to have a much higher Young's modulus, which was ∼2–3 orders of magnitude higher than those of other FsLDW-fabricated protein microstructures^27^, both in the dry and wet states (see Fig. 2e,f, Supplementary Fig. 4 and, for details, Supplementary Discussion). On the basis of the deflection–displacement loading force curves in Fig. 2e, Young's moduli of the RSF microsquare dried in air (∼2.2 GPa) and equilibrated in water (∼0.22 GPa) could be calculated using the Hertz model (see details in Methods)^32^. The much higher Young's moduli of FsLDW-polymerized RSF might provide great help and options for adjusting mechanical characteristics of protein-based composite materials (with ‘soft' proteins^27^) processed by FsLDW multiphoton lithography, aiming at different needs from various biomedical applications, especially such as bone cell culturing and tissue engineering^1^,^2^.

### Silk/Ag composite FsLDW

Owing to various exploitable chemical groups, multilevel 3D conformation and resulted properties of RSF, RSF-centred aqueous ink could be ‘configurable' and ‘modularized' for diverse functionalization^1^,^2^,^28^,^29^,^30^. Here in aqueous ink (Fig. 1ciii), RSF crosslinking and Ag^+^ photoreduction might occur simultaneously under high-intensity femtosecond laser irradiation (FsLDW parameters listed in Methods). RSF behaved as a bioreductant^30^,^33^ and crosslinkable biopolymer matrix in the fabrication process, and silk/Ag composite micro/nanostructures were FsLDW-constructed from RSF/AgNO_3_ aqueous solutions (Fig. 1d,f). Generally, the ‘window' of applicable FsLDW parameters was limited, and usually the optimal parameters should be adopted for high fabrication quality. Therefore, it might be difficult to tailor content ratios of components in polymer/metal composite micro/nanostructures by changing the FsLDW-processing parameters. However, in our case, the Ag content ratio in crosslinked RSF matrices could be facilely controlled by changing metallic ion concentrations^26^ or pre-reducing/-loading Ag nanoseeds^30^. This multidimensional controllability of silk-based FsLDW multiphoton lithography, that is, not only arbitrarily desgined geometries but also tailorable composition and functions of obtained micro/nanostructures (so-called ‘multiple arbitrary customizations'), is valuable for its wider application.

**Figure 3:**
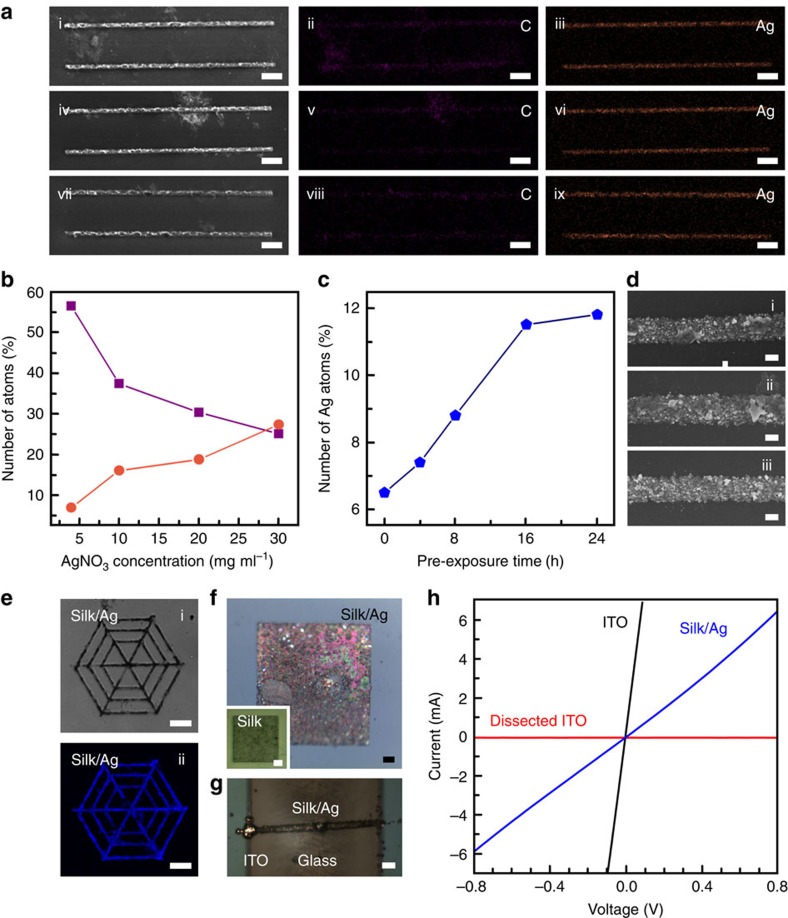
Silk/Ag composite micro/nanostructures/devices customized by FsLDW multiphoton lithography. (**a**)i,iv,vii) SEM images of silk/Ag composite microwires; (ii,v,viii) EDS distribution maps of elemental C of silk/Ag composite microwires; (iii,vi,ix) EDS distribution maps of elemental Ag of silk/Ag composite microwires. Scale bars, 10 μm. RSF concentration was constant at 2.5 wt%; AgNO_3_ concentrations were 10 mg ml^−1^ for (i–iii), 20 mg·ml^−1^ for (iv–vi) and 30 mg·ml^−1^ for (vii–ix). (**b**) Atom number ratios in FsLDW-fabricated silk/Ag composite versus different AgNO_3_ concentrations in RSF/AgNO_3_ inks. Violet squares, C; orange rounds, Ag. (**c**) Ag atom number ratios in silk/Ag composite FsLDW-fabricated from RSF/AgNO_3_/Ag nanoseed inks with different pre-exposure times. (**d**) SEM images of silk/Ag composite microwires FsLDW-fabricated from RSF/AgNO_3_/Ag nanoseed inks with different pre-exposure times ((i) 0 h; (ii) 8 h; (iii) 16 h). Scale bar, 1 μm. (**e**) Confocal microscopic images of a silk/Ag composite ‘microcobweb'. (i) The bright-field image; (ii) the dark-field 405-nm-excited fluorescent image). Scale bars, 10 μm. (**f**) The metallographic OM image of a FsLDW-fabricated silk/Ag composite microsquare. Inset, the metallographic OM image of a FsLDW-fabricated all-silk-based microsquare. Scale bars, 10 μm. (**g**) The metallographic OM image of a FsLDW-fabricated silk/Ag composite microwire between two ITO electrodes. Scale bar, 10 μm. (**h**) Current–voltage curves of the silk/Ag composite microwire in **g**, continuous ITO film and ITO film dissected into two ITO electrodes.

In Fig. 3a, after water rinsing, silk/Ag composite microwires with ∼2-μm width and 100-μm length were directly written out on glass substrates, and their morphologies were not affacted by different Ag^+^ concentrations in RSF/AgNO_3_ aqueous inks. Using energy-dispersive spectrometer (EDS) measurement, the distribution maps of elements mainly including Ag, carbon (C) and nitrogen (N) suggested that C (Fig. 3aii,v,viii) and Ag (Fig. 3aiii,vi,ix) distributed in good correspondence with silk/Ag composite microstructures in SEM images (Fig. 3ai,iv,vii). It indicates that silk crosslinking and metal photoreduction simultaneously happened in Fs-laser-scanned regions. Along with the increase of AgNO_3_ concentration in RSF/AgNO_3_ aqueous inks in Fig. 3b, silk/metallic Ag composite showed increasing Ag and decreasing C atom number ratios (versus atom sum of Ag, C and N). When the AgNO_3_ concentration was changed from 4 to 30 mg ml^−1^, Ag atom number ratio rised correspondingly from ∼7 to 27%.

More importantly, content of metallic Ag can also be well controlled through pre-reducing Ag^+^ and therefore pre-loading Ag nanoseeds in RSF under incandescent light or sunlight^30^ before FsLDW (see Supplementary Fig. 5). During this pre-exposure, Ag^+^ can be reduced by oxidizable groups of RSF such as Tyr to produce metallic Ag nanoseeds in RSF^30^,^33^. As proved previously, the limited mobility made Ag^+^ quickly exhausted in the laser spot region during FsLDW^34^,^35^. However, the long-time photo-pre-reduction of Ag^+^ does not meet such a problem. As a pre-enrichment, much more Ag^+^ in the whole solution can be reduced under long-time pre-exposure^30^,^33^, which dramatically raised the metallic Ag content in FsLDW-fabricated silk/Ag composite micro/nanostructures.

In a representative experiment, the aqueous solution with 2.5 wt% RSF and 4-mg ml^−1^ AgNO_3_ was pre-exposed under a 40-W incandescent lamp for 0–24 h (h) or longer (see Supplementary Fig. 6). Ultraviolet-visible absorption spectra of pre-exposed RSF/AgNO_3_ inks in Supplementary Fig. 6a showed increasing peaks with increasing pre-exposure time, corresponding well to the gradually deepening solution colours (Supplementary Fig. 6b). These results demonstrate that more Ag nanoseeds formed after a longer time pre-exposure^30^. Consequently, as displayed in Fig. 3c, the Ag content ratio in FsLDW-fabricated silk/Ag composite was increased by increasing the pre-exposure time but tended to be saturated until 16 h. This was consistent with the reported ‘threshold' pre-exposure time (∼20 h) for saturated Ag reduction with similar experimental set-ups^30^. Compared with FsLDW using the original ink containing 2.5 wt% RSF and 4-mg ml^−1^ AgNO_3_, Ag atom number ratio in Ag, C and N of FsLDW-fabricated silk/Ag composite obviously increased from ∼6.5% (0-h pre-exposure) to 12% (24-h pre-exposure) by almost two times (Fig. 3c). Meanwhile, as shown in Fig. 3d, there was no obvious difference in morphology among silk/Ag composite microstructures with varying-degree pre-exposure. The reason is probably that the Ag nanoseeds produced via varying pre-exposure time have similar diameter distribution^30^ (∼14–20 nm; see Supplementary Fig. 7), which was also evidenced by unchanged peak wavelength (∼440 nm) of absorption spectra of different samples (Supplementary Fig. 6a).

After comprehensive exploration of silk/Ag composite FsLDW, various silk/Ag composite micro/nanodevices can be facilely written out (see Fig. 3e–g). In confocal microscopic images of Fig. 3e, a silk/Ag-based microscale ‘spider web' was FsLDW-fabricated on a glass slice, showing 405-nm-excited blue fluorescence from polymerized RSF (Fig. 3eii), similar to all-RSF micro/nanostructures mentioned above. In metallurgical OM images in Fig. 3f, a FsLDW-fabricated silk/Ag composite microsquare (100 × 100 × 2 μm^3^) had a shiny metallic lustre that was not shown from a RSF-based microsquare (50 × 50 × 2 μm^3^) in the inset. This was a complementary and apparent indication of the considerable production and content of metallic Ag in silk/Ag multiphoton-absorption (MPA) FsLDW and obtained micro/nanostructures, respectively. Furthermore, we directly ‘wrote' a prototyping electroconductive (∼8.47 × 10^4 ^Ω^−1 ^m^−1^) silk/Ag microwire out of originally insulated RSF between two unconnected indium tin oxide (ITO) electrodes parallelly on a glass substrate (Fig. 3g; see details in Methods, Supplementary Fig. 8 and Supplementary Methods). Significantly, we could facilely adjust the electric conductivity of silk/Ag microwires by changing concentrations of ink components and consequent metallic Ag content in FsLDW-fabricated silk/Ag composites (see Supplementary Fig. 9 and Supplementary Fig. 10 and, for details, Supplementary Methods).

### Silk/Au composite FsLDW

**Figure 4:**
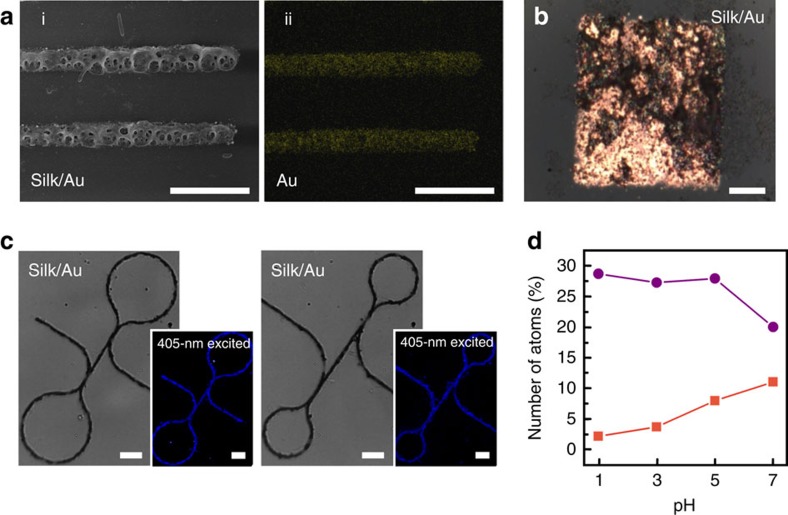
Silk/Au composite micro/nanostructures customized by FsLDW multiphoton lithography. (**a**i) SEM image of silk/Au composite microwire; (ii) EDS distribution maps of elemental Au of silk/Au composite microwires; scale bars, 10 μm. (**b**) The metallographic OM image of a FsLDW-fabricated silk/Au composite microsquare. Scale bars, 10 μm. (**c**) Confocal microscopic images of silk/Au composite micropatterns. Insets, dark-field 405-nm-excited fluorescent images. Scale bars, 10 μm. (**d**) Atom number ratios in FsLDW-fabricated silk/Au composite versus different pH values in RSF/HAuCl_4_ inks. Violet rounds, C; orange squares, Au.

As a versatile protein-based platform, RSF can also be applied for FsLDW fabrication of silk/Au composite micro/nanodevices (Fig. 1g and Fig. 4) reasonably via a similar mechanism as preceding silk/Ag composite FsLDW. It was found that relatively high concentrations of RSF and HAuCl_4_ were easy to lead into self-gelation of RSF/HAuCl_4_ aqueous solutions, excessively active photochemical reactions of Au reduction by RSF and consequent poor control of FsLDW processing. Therefore, during FsLDW using RSF/HAuCl_4_ aqueous solutions, lower concentrations of both RSF and HAuCl_4_ were crucial, and optimal FsLDW-processing parameters were similar with those of silk/Ag composite FsLDW because of the limited ‘parameter window' (see FsLDW parameters in Methods). A variety of fine silk/Au composite micropatterns were FsLDW-fabricated on glass substrates as shown in Figs 1g and 4. In Fig. 4a, SEM images of silk/Au composite microwires (Fig. 4ai) matched well with elemental distribution maps of Au (Fig. 4aii), C and N (Supplementary Fig. 11), indicating the simultaneous silk photocrosslinking and Au photoreduction in Fs-laser-irradiated regions. An as-formed silk/Au composite microsquare (40 × 40 × 2 μm^3^) also shows a bright golden lustre under a metallurgical OM (see Fig. 4b). In addition, in confocal images of Fig. 4c, obvious 405-nm-excited fluorescence was emitted probably from RSF in arbitrary silk/Au composite micropatterns constructed by FsLDW multiphoton lithography.

Interestingly, metallic Au content in FsLDW-fabricated silk/Au composite micro/nanostructures can be facilely controlled by merely changing the pH value of RSF/HAuCl_4_ aqueous inks. When solution pH values were changed from 1.0 to 7.0, Au atom number ratio in Au/C/N atom sum of FsLDW-fabricated silk/Au composite increased from ∼2.3 to 11.1% along with C content variation from ∼28.7 to 20.0% (Fig. 4d). It relied on that more phenolic groups of Tyr residues in RSF would be ionized in solution with higher pH value to facilitate the electron transfer from Tyr residues to trivalent Au (refs 29, 33). Thus, the pH-dependent facile adjustments of metallic Au content in FsLDW-fabricated silk/Au composite can be well achieved without obvious morphologic fluctuations of obtained micro/nanostructures (see Supplementary Fig. 12).

### Silk-centred FsLDWs for biorelated applications

For probable biorelated applications, biocompatibility of SF micro/nanostructure FsLDW-fabricated and SF-centred inks used was evaluated (see details in Supplementary Discussion). As shown in Supplementary Fig. 13, the cells (mouse fibroblast cell line, L929) showed normal morphology after 1-day incubation. Cell viability was high for all-silk-centred microwire or microdot arrays (all-silk micropatterns with a little MB remained, ∼90% cell viability; silk/Ag micropatterns, ∼105%; silk/Au micropatterns, ∼101%; see Supplementary Fig. 14). All FsLDW-fabricated silk-centred microstructures owned fairly good biocompatibility, which is of great facilitation for their utilization in bioelectrical researches and applications (for example, micro/nano-level testing and manipulating nerve cells and even bioelectric signals) and bio-MEMS.

Remarkably, together with mask-free and noncontact features, direct-writing-mode multiphoton lithography can be endowed with a better simplification and applicability for complex processing environments and small-batch customization (especially valuable for biorelated micro/nanofabrication). By using silk/MB or silk/[AuCl_4_]^−^ inks with confirmed acceptable biocompatibility (Supplementary Fig. 15), we further demonstrated the feasibility of ‘*in situ*' aqueous FsLDW where live bacteria (Supplementary Fig. 16, Supplementary Movie 1, Supplementary Movie 2 and Supplementary Movie 3) or cells (Supplementary Fig. 17, Supplementary Movie 4 and Supplementary Movie 5) were close to the laser-processing microareas (see details in Supplementary Discussion). On the basis of its inherent high precision, low collateral thermal damage, noncontact feature, flexibility and facile designability, Fs-laser processing would not induce significant impact on live bacteria or cells nearby. It might have great potential for direct micro/nanofabrication/operation in complex bioenvironments (for example, micro/nano-level ‘live' (real-time) and ‘*in situ*' manipulating bacteria or cells, and especially, stimulating or testing a single nerve cell), which is fairly difficult to implement via other micro/nanophoto-processing technologies.

### ATR-FTIR characterization of FsLDW-fabricated RSF

**Figure 5:**
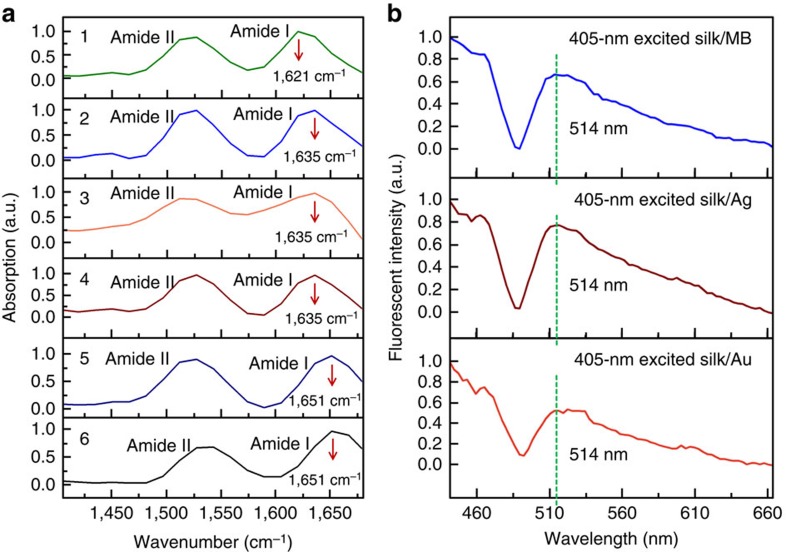
ATR-FTIR and fluorescent spectrum analyses of various RSF-based samples. (**a**) ATR-FTIR absorption spectra of various RSF-based samples. (**b**) Fluorescence-emitting spectra of 405-nm-excited all-silk-based, silk/Ag and silk/Au FsLDW-fabricated microstructures.

To confirm the conformations of FsLDW-polymerized RSFs, attenuated total reflection Fourier transform infrared (ATR-FTIR) spectra of different RSF-based samples were obtained as shown in Fig. 5a (see details in Methods). Amide-I absorption peaks of various FsLDW-fabricated RSF-based samples were located at ∼1,635 cm^−1^ (Fig. 5a 2–4), in between 1,621 cm^−1^ of a completely β-sheet-dominated RSF film (Fig. 5a1) and 1,651 cm^−1^ of amorphous RSF films (Fig. 5a5)^6^,^11^. More importantly, it was found that indentation loading curves and Young's moduli were almost unchanged for the FsLDW-fabricated RSF-based microsquare (20 × 20 × 4 μm^3^, tested in Fig. 2e) before and after 2 min immersion in 70% (v/v) ethanol aqueous solution, which is a well-known solution to transfer the random coil conformation of RSF to the β-sheet^18^. Therefore, it suggested that RSF might be already β-sheet-crystallized during FsLDW MPA polymerization probably by heating and photo-stimulations of Fs laser. It should be noted that the infrared absorption peak of various FsLDW crosslinked RSFs is at 1,635 cm^−1^, which was assigned to the β-sheet conformation (red shifted ∼14 cm^−1^ from 1,621 cm^−1^ of the ethanol-treated RSF film)^1^,^2^,^6^. The reason might be that the conformation transition of FsLDW-crosslinked RSFs was prevented by covalent photocrosslinking of oxidizable groups such as Tyr in an uncompleted way. Importantly, in Supplementary Fig. 18, infrared absorption ∼1,050–1,150 cm^−1^ strongly demonstrated more formation of C–O–C bonds, implying Tyr-involved oxidation crosslinking of RSF during FsLDW^36^.

### Fluorescence of FsLDW-fabricated RSF-based microstructures

Under 405-nm excitation, approximately blue fluorescence from FsLDW-fabricated RSF micro/nanostructures was observed with naked eyes. Accordingly, computer false colours were adopted for corresponding fluorescent OM and confocal microscopic images (Figs 2b,c, 3e and 4c). Via confocal microscopic characterization in Fig. 5b, fluorescent spectra of FsLDW-fabricated RSF-centred microstructures (all-RSF, silk/Ag and silk/Au) were similar under 405-nm excitation. Therefore, the fluorescence should be intrinsically emitted from RSF in FsLDW-fabricated microstructures. Actually, SF itself originally has the photoluminescence phenomenon, for instance, a 305-nm-stimulated fluorescent peak at 340 nm (ref. 37). Ultraviolet-processed RSF exhibited enhanced fluorescence and even new peaks emerged in the 400∼470-nm region (305-nm excitation)^37^. Correspondingly, 405-nm-excited FsLDW-fabricated microsilks emitted enhanced fluorescence in the 500-nm region (∼514 nm) in our work. Photoproducts and crosslinks formed during photoprocessing might bring new chromophores to cause the fluorescence change of RSF (more discussion in Supplementary Discussion 8)^37^. Especially, dityrosine crosslinks emitted fluorescence ∼400 nm under 305-nm excitation (correspondingly, 500-nm fluorescence under 405-nm excitation in Fig. 5b), which was considered to be related to the fluorescent changes of RSF after Ultraviolet or 800-nm Fs-laser processing^37^. Namely, the changed fluorescence is also a clue indicating probable Tyr-involved covalent crosslinking of RSF during FsLDW.

### Probable mechanisms of silk-centred FsLDW

The focal temperature might be ∼70–100 °C in silk-centred inks during FsLDW with respective optimal processing parameters (see detailed data and discussion in Supplementary Discussion 8 and Supplementary Movie 6–11). This temperature range could induce significant β-crystallization of RSF, which could be confirmed by the amide-I absorption peak at 1,635 cm^−1^ in ATR-FTIR spectra (Fig. 5a). Namely, photothermal effect probably also played an important role in micro/nanocuring RSF during FsLDW. On the other hand, the mechanism has been widely accepted and applied to various proteins' FsLDW that enhanced production of singlet oxygen via two-photon MB photosensitization (^1^O_2_, or other oxidizing species by other photosensitizers) promoted protein crosslinking under high-intensity irradiation of infrared femtosecond laser pulses^38^,^39^. Series of existing researches have also experimentally demonstrated that light-irradiated nanometals did enhance localized electromagnetic field^34^, photothermal effects^40^,^41^ (also proved in our experiments presented in Supplementary Discussion and Supplementary Figs 19–22) and photo-production of ^1^O_2_ (or other oxidizing species)^42^. Therefore, photo-oxidation crosslinking in our RSF system was believed to be probably also ‘catalysed' by MB or nanometals. In the experiments, we found that FsLDW-fabricated SF microstructures could not be totally hydrolysed and dissolved in 60-°C-dense LiBr aqueous solution (efficiently breaking hydrogen bonds and dissociating β-sheets to dissolve β-conformation SF, even raw silk fibres; Supplementary Fig. 23). Especially, both ATR-FTIR absorption at ∼1,050–1,150 cm^−1^ (Supplementary Fig. 18) and 500-nm-region fluorescence (405-nm excitation; Fig. 5b) of FsLDW-fabricated microsilks strongly indicated the Tyr-involved crosslinking of RSF during FsLDW^36^,^37^. In addition, as a reasonable and frequently used parallel of 800-nm infrared multiphoton process, the time-resolved investigation of ultraviolet processing also suggested the formation of both β-sheets and covalent crosslinks in 405-nm-exposed silk/AgNO_3_ inks. Therefore, both Fs-laser-induced oxidation crosslinking and β-folding involved and resulted in RSF micro/nanocuring, and consequently, the silk-centred FsLDW micro/nanofabrication here (see detailed discussion in Supplementary Discussion 8 and Supplementary Figs 23–29).

## Discussion

In summary, we reported versatile aqueous multiphoton lithography using diversiform ‘modularized' *Bombyx mori* SF-centred inks via noncontact and maskless FsLDW. First, without any chemical modification, arbitrary fine 2D/3D micro/nanostructures were readily FsLDW-fabricated from RSF ink merely with the help of photosensitizer MB. The RSF-based micro/nanostructure exhibited remarkable mechanical characteristics to prevent collapse, mainly because of the β-sheet structure in RSF, which was preliminarily proved using ATR-FTIR^6^,^11^. It might bring significant convenience for tuning mechanical properties of FsLDW-realized protein-based composite for its fine applications^1^,^2^. Further, RSF functioned actively as reductant in silk/Ag^+^ and silk/[AuCl_4_]^−^ aqueous inks, and became a biopolymer matrix to FsLDW-customize silk/metal composite micro/nanodevices via simultaneous RSF photocrosslinking and metal photoreduction. Without changing FsLDW parameters already optimized, the metal content in as-formed silk/metal composite micro/nanostructures could be well controlled facilely and multidimensionally by either changing metallic ion concentrations or pre-loading Ag nanoseeds on RSF, or even adjusting pH values of inks. Silk/Ag microwires exhibited adjustable 10^4^-Ω^−1 ^m^−1^-scale electric conductivity. Thus, the aqueous FsLDW multiphoton lithography with multifunctional silk-based bioresists displayed ‘multiple arbitrary customizations' of material composition, 3D structure geometries and fine biocompatibility of both obtained silk-centred microdevices and processing environments. New opportunities might be opened for fields of silk-based micro/nano-level electronic and mechanical bioengineering and biosystems in the future, for example, micro/nanoscale bioelectric stimulation and test of a nerve cell, ‘live' (real-time) and ‘*in situ*' cell micro/nanomanipulation.

## Methods

### Preparation of RSF aqueous mother solution

Silk consists of fibroin fibres that are bound together by sericin, which are hydrophilic gum-like coat proteins. The degumming (removing the sericin) and dissolving process of SF fibre followed procedures reported previously^30^,^31^. Then, the SF solution was dialysed against deionized water for 72 h at room temperature with a semipermeable membrane (MEMBRACEL, 12,000–14,000 molecular weight cut off) to remove LiBr. The dialysed SF solution was centrifuged at 6,000 r.p.m. for 5 min, and the supernatant was collected at room temperature and stored at 4 °C. The concentration of the final SF solution was ∼3 wt%.

### Preparation of RSF-based aqueous FsLDW inks

All the inks in this work were prepared using ultrapure water (18.2 MΩ cm, 25 °C) from a MILLIPORE water purification system. By mixing 3 wt% RSF mother solution and 1 mg ml^−1^ MB mother solution (v/v, 1 ml: 0.2 ml), the RSF-based aqueous inks were prepared (RSF ∼2.5 wt%, MB 0.17 mg ml^−1^).

The RSF/AgNO_3_ aqueous inks were prepared by blending 3 wt% RSF mother solution and different AgNO_3_ mother solutions as follows:

1, 2-ml RSF solution (3 wt%)+0.4-ml AgNO_3_ solution (24 mg ml^−1^)=RSF ∼2.5 wt%, AgNO_3_ 4 mg ml^−1^;

2, 2-ml RSF solution (3 wt%)+0.4-ml AgNO_3_ solution (60 mg ml^−1^)=RSF ∼2.5 wt%, AgNO_3_ 10 mg ml^−1^;

3, 2-ml RSF solution (3 wt%)+0.4-ml AgNO_3_ solution (120 mg ml^−1^)=RSF ∼2.5 wt%, AgNO_3_ 20 mg ml^−1^;

4, 2-ml RSF solution (3 wt%)+0.4-ml AgNO_3_ solution (180 mg ml^−1^)=RSF ∼2.5 wt%, AgNO_3_ 30 mg ml^−1^;

5, 1-ml RSF solution (3 wt%)+1-ml AgNO_3_ solution (300 mg ml^−1^)=RSF ∼1.5 wt%, AgNO_3_ 150 mg ml^−1^ (for the conductive silk/Ag microwire).

The RSF/HAuCl_4_ aqueous inks (RSF, 0.2 wt%; HAuCl_4_, 0.33 mg ml^−1^) were prepared by mixing 0.3 wt% RSF solution and 1-mg ml^−1^ HAuCl_4_ solution by 2:1 (v/v). Then, the inks were titrated with HCl aqueous solution (10 mol l^−1^) and NaOH aqueous solution (10 mol l^−1^) to pH values (1.0, 3.0, 5.0 and 7.0), as needed.

### Pre-exposure of RSF/AgNO_3_ inks for FsLDW

Being sealed in transparent polystyrene centrifuge tubes, RSF/AgNO_3_ aqueous solutions (RSF ∼2.5 wt%, AgNO_3_ 4 mg ml^−1^) were placed 3 cm under the 40-W incandescent lamp for different time as needed (0–24 h or more at 25 °C).

### FsLDW experimental procedures

For multiphoton lithograhpy, the femtosecond laser (Spectra Physics 3960-X1BB, 80-MHz repetition rate, 120-fs pulse width, 800-nm central wavelength) beam was tightly focused in silk-based aqueous resists using a high-numerical-aperture oil-immersion objective lens (Olympus, × 60, numerical aperture=1.35). Laser power was measured before the objective, and the two-minor-axis cross-section of ellipsoidal focal spot was ∼0.4 μm^2^ to estimate the laser power density. 3D scanning of the focused laser spot and therefore 3D micro/nanofabrication were achieved based on the cooperation of a two-galvano-mirror set (horizontal movements) and a piezo stage (Physik Instrumente P-622. ZCD, vertical movements). The geometries of micro/nanostructures/devices were designed by 3Ds Max to obtain corresponding computer-processing data. (For instance in Fig. 2d: (i) 3D microbowl, bottom diameter, 12 μm; top diameter, 15 μm; height, 6 μm; wall thickness, 1 μm. (ii) Another 3D microbowl, bottom diameter, 15 μm; top diameter, 12 μm; height, 4 μm; wall thickness, 1 μm. (iii) An impending microwire, microwire diameter, ∼760 nm. (iv) A microscale frustum of a pyramid, bottom, 5 × 5 μm^2^; top, 2.5 × 2.5 μm^2^; height, 3 μm.) Controlled by the processing data, various complicated micro/nanostructures/devices would be readily fabricated on the substrates by FsLDW multiphoton lithography after water rinsing.

### FsLDW parameters for different cases

For all the RSF/MB inks, optimal FsLDW parameters were ∼75.0-mW μm^−2^ laser power intensity, 100-nm scanning step and 1,000-μs exposure time on single point. For all the RSF/Ag^+^ inks and the RSF/[AuCl_4_]^−^ inks, optimal FsLDW parameters were ∼12.5-mW μm^−2^ laser power intensity, 100-nm scanning step and 1,000-μs exposure time on single point.

### Young's modulus determination with the Hertz model

The indentation loading deflection–displacement curves in Fig. 2e and Supplementary Fig. 4c were obtained by using a Veeco MultiMode-8 AFM system (contact modes in air and in water as needed) and a silicon nitride cantilever from Veeco (MLCT-AUNM). The data were analysed with the Nanoscope-8.10 software. The parameters of the silicon nitride cantilever were listed in Supplementary Fig. 4a, where the spring constant (*k*) of the cantilever was determined to be ∼42 N m^−1^ via the thermal noise method^32^.

On the basis of the deflection–displacement curves, Young's moduli of FsLDW-fabricated RSF-based microstructures dried in air and immersed in water were calculated via the Hertz model as follows^32^:


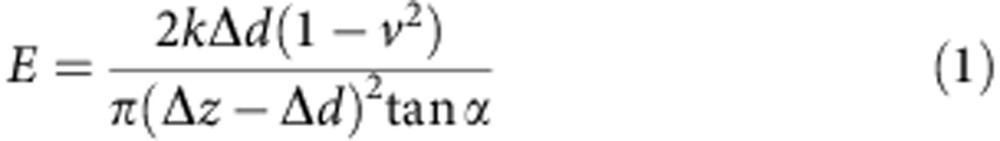


and Δ*d=d*_e_–*d*_c_, Δ*z=z*_e_–*z*_c_,

among which *E* is Young's modulus, *v* is Poisson's ratio (*v*=0.5), *α* is the opening angle of the cantilever-tip cone (*α*=20.6° as shown in Supplementary Fig. 4b), *d*_c_ and *z*_c_ are the cantilever deflection and piezo displacement of the contact point, and *d*_e_ and *z*_e_ are the cantilever deflection and piezo displacement of the end point.

### ITO electrode preparation and electroconductivity tests

The ITO film was ∼100-nm thick, and the sheet resistance was ∼16.5 Ω. An ∼100-μm-wide gap of the ITO film was ablated to obtain two ITO electrodes by the pulse-amplified femtosecond laser (1-kHz repetition rate, 100-fs pulse width, 800-nm central wavelength; focused with a convex lens of 625-mm focal length; average power, 100 mW; scanning speed, 2 mm s^−1^).

Current–voltage curves of the silk/Ag composite micowire were measured with a Keithley 2400 programmable voltage–current characterization system. During the testing processes, electrode holders connected with the sample were fixed as a whole to avoid errors resulted by connection change of electrodes.

### ATR-FTIR characterization and sample preparation

Microscopic ATR-FTIR characterization was performed using the NICOLET 6700 FTIR system equipped with a NICOLET CONTINUμM FTIR microscope. For sample preparation, 1-ml 3 wt% RSF aqueous solution was dripped on a glass slide to spread out and form a thick film after 5 min drying in air (∼25% relative humidity, 25 °C; for Fig. 5 a-5) or 5 min heating and drying (95 °C; for Fig. 5 a-4). Then, the standard samples of amorphous RSF films were prepared. The completely β-pleated sheet-crystallized RSF film was prepared by immersing the air-dried amorphous RSF film in 70% (v/v) ethanol aqueous solution for 2 min (Fig. 5 a-1).

### Other characterizations

OM images were obtained with a BA310Met metallographic microscope equipped with the charge-coupled device. SEM characterization was performed using a field emission SEM (JSM-7500F, JEOL), and the samples were sputter-coated with an Au film (Pt film for silk/Au samples) with a thickness of 4–5 nm in vacuum using an auto fine coater (JFC-1600, JEOL). Topography of RSF-centred micro/nanostructures was AFM-characterized in air with the tapping mode using Veeco NanoScope V. EDS analysis was carried out with the AMETEK APOLLO-XL EDS system integrated with the SEM system. Confocal microscopic characterization was performed via the OLYMPUS FLUOVIEW-FV1000 confocal microscope. Ultraviolet-visual spectra were obtained with the SHIMADZU UV-2550 ultraviolet and visible spectrophotometer. Transmission electron microscopy (TEM) images were taken with a HITACHI Mic-H-600 TEM system.

## Additional information

**How to cite this article:** Sun, Y.-L. *et al*. Aqueous multiphoton lithography with multifunctional silk-centred bio-resists. *Nat. Commun.* 6:8612 doi: 10.1038/ncomms9612 (2015).

## Supplementary Material

Supplementary Information Supplementary Figures 1-29, Supplementary Methods, Supplementary Discussion and Supplementary References

Supplementary Movie 1 "In-situ" FsLDW fabricating a micro-triangle in silk/MB ink containing live bacteria

Supplementary Movie 2 "In-situ" FsLDW fabricating a micro-circle in silk/MB ink containing live bacteria.

Supplementary Movie 3 "In-situ" FsLDW fabricating a micro-wire in silk/[AuCl4] - ink containing live bacteria

Supplementary Movie 4 "In-situ" FsLDW fabricating a micro-square around a Hela cell in silk/MB ink.

Supplementary Movie 5 "In-situ" FsLDW fabricating a micro-circle around a Hela cell in silk/[AuCl4] - ink.

Supplementary Movie 6 FsLDW in silk/MB ink with Fs-laser power intensity of ∼ 75 mW·μm^-2^ (measured before the objective lens).

Supplementary Movie 7 FsLDW in silk/[AuCl4] - ink with Fs-laser power intensities of ∼ 2.1 mW·μm^-2^ (measured after the objective lens).

Supplementary Movie 8 FsLDW in silk/[AuCl4] - ink with Fs-laser power intensities of ∼ 3.5 mW·μm^-2^ (measured after the objective lens).

Supplementary Movie 9 FsLDW in silk/AgNO3 ink with Fs-laser power intensities of ∼ 3.5 mW·μm^-2^ (measured after the objective lens).

Supplementary Movie 10 FsLDW in silk/AgNO3 ink with Fs-laser power intensities of ∼ 5 mW·μm^-2^ (measured after the objective lens). Movie 11 | Fs-laser was focused in pure SF

Supplementary Movie 11 Fs-laser was focused in pure SF aqueous solution for seconds (∼ 75 mW·μm^-2^ before the objective lens).
