# Supplementary material for: Aqueous multiphoton lithography with multifunctional silk-centred bio-resists
Source: Nat Commun. 2015 Oct 16;6:8612. doi: 10.1038/ncomms9612 (PMC4634322; doi:10.1038/ncomms9612)
Supplement: Supplementary Information — Supplementary Figures 1-29, Supplementary Methods, Supplementary Discussion and Supplementary References [file ncomms9612-s1.pdf]

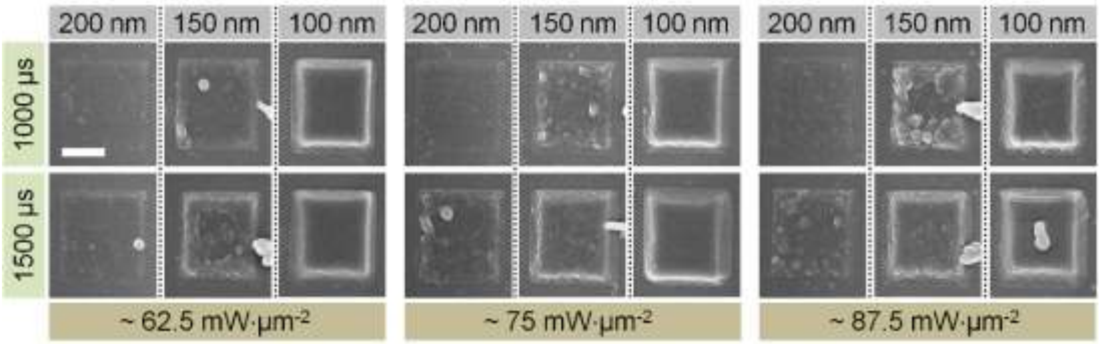

Supplementary Figure 1 | Optimization of FsLDW-processing parameters. Laser power intensity from about 62.5 to 87.5 mW·μm^{-2}; scanning step from 100 nm to 200 nm; exposure time on single point, 1000 and 1500 μs. Scale bar, 5 μm.

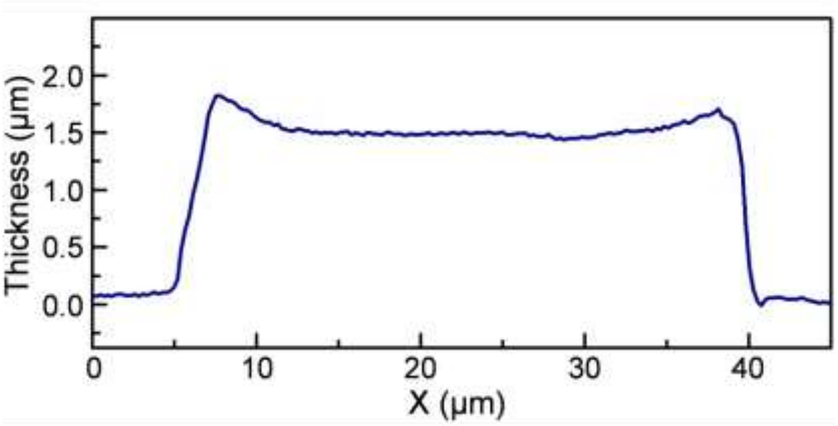

Supplementary Figure 2 | The cross-section profile of the all-silk-based micro-hexagon in Figure 2b of main text by AFM characterization.

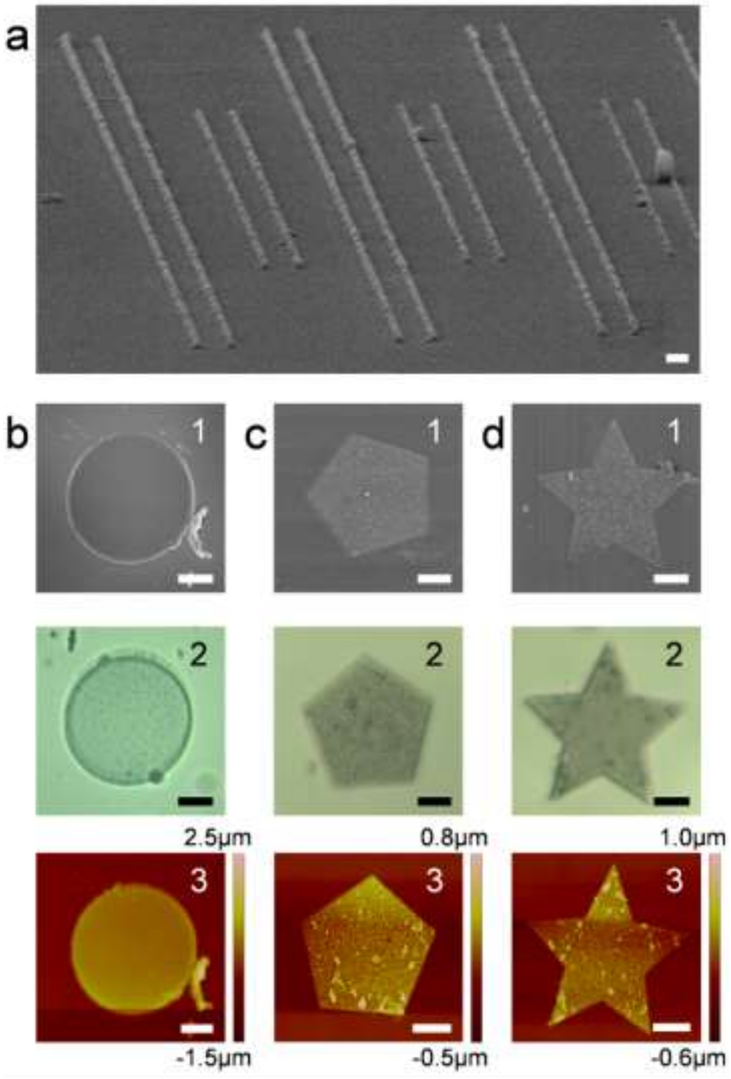

Supplementary Figure 3 | All-silk-based micro/nano-structures FsLDW-fabricated with various geometries. (**a**) The oblique-view SEM image of all-silk-based nanowires in Figure 2a of main text. Scale bar, 1 μm. (**b**) An all-silk-based micro-round;1, the SEM image; 2, the OM image; 3, the AFM graph exhibiting about 14-nm Ra; scale bars, 10 μm. (**c**) An all-silk-based micro-pentagon;1, the SEM image; 2, the OM image; 3, the AFM graph exhibiting about 66-nm Ra; scale bars, 10 μm. (**d**) An all-silk-based five-pointed micro-star; 1, the SEM image; 2, the OM image; 3, the AFM graph exhibiting about 60-nm Ra; scale bars, 10 μm.

a

Lever					Tip	
Thickness (μm)	Length (μm)	Width (μm)	Spring const. (N/m)	Resonant freq. (kHz)	Height (μm)	Radius (nm)
4.6	160	50	42 (12-103)	300 (200-400)	11 (7-15)	<10

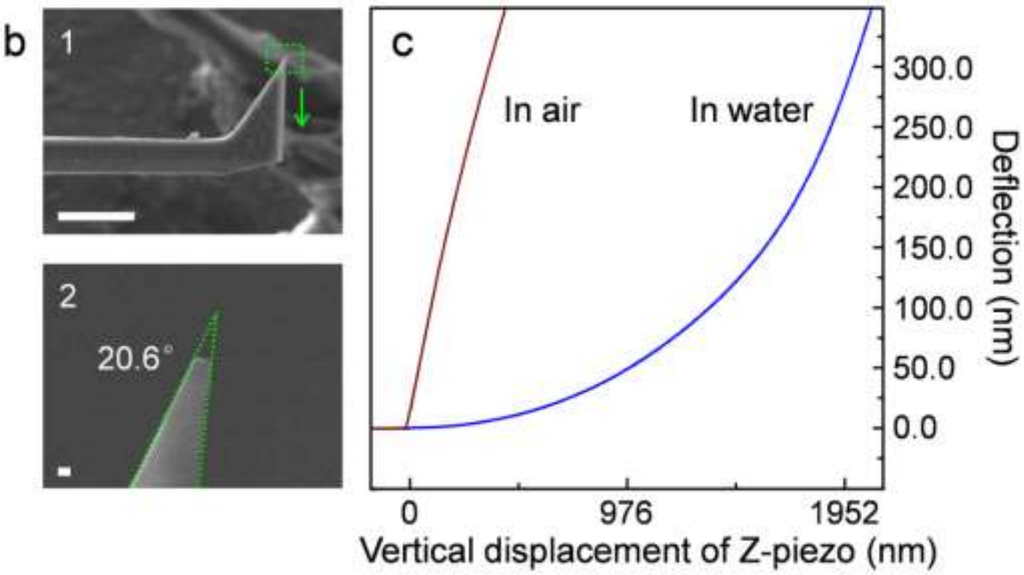

Supplementary Figure 4 | (a) Parameters of the silicon nitride cantilever used for Young's modulus determination. (**b**) SEM images of the silicon nitride cantilever. Scale bars: 1, 10 μm; 2, 1 μm. (**c**) Large-scope indentation loading deflection-displacement curves of an all-silk-based micro-square dried in air and equilibrated in water, corresponding to Figure 2e of main text.

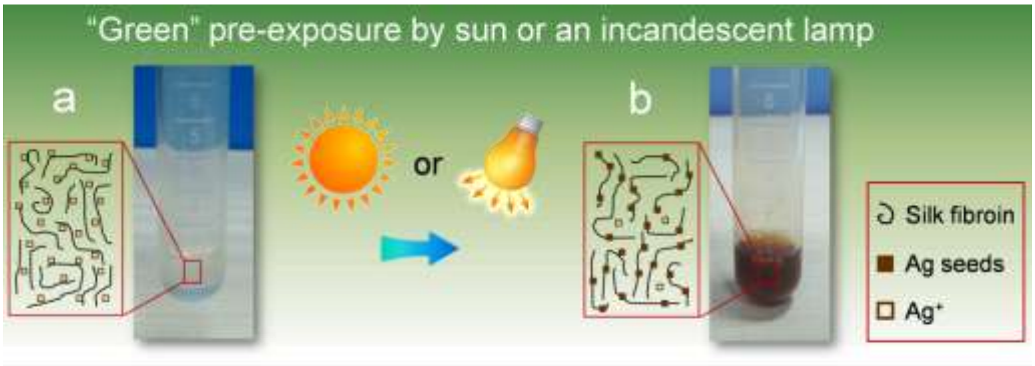

Supplementary Figure 5 | "Green" pre-reduction and pre-loading of Ag nano-seeds on RSF by pre-exposure of light sources like incandescent light and sunlight before FsLDW. (**a**) RSF/$AgNO_3$ aqueous solution before pre-exposure. (**b**) RSF/Ag-nanoseeds aqueous solution after pre-exposure.

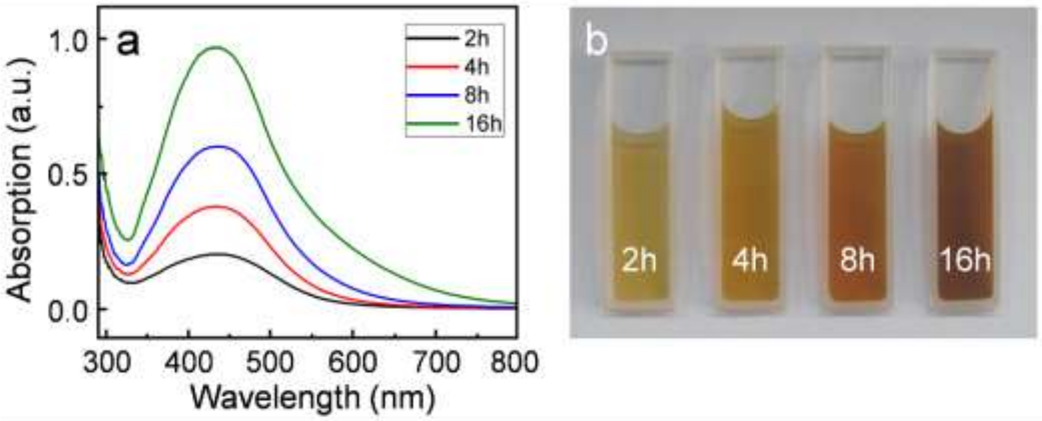

Supplementary Figure 6 | (**a**) UV-visual absorption spectrums of RSF/$AgNO_3$ aqueous inks pre-exposed for from 2 to 16 hours. (**b**) Images of RSF/$AgNO_3$ aqueous inks pre-exposed for from 2 to 16 hours.

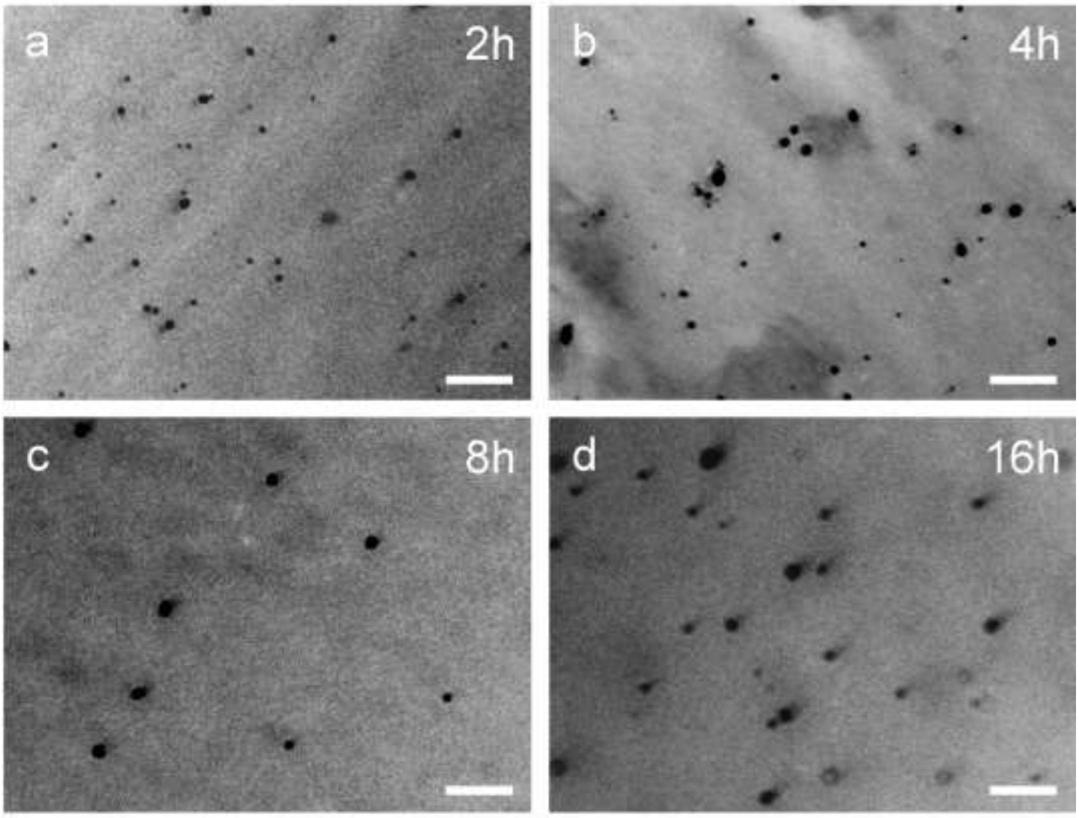

Supplementary Figure 7 | TEM images of Ag nano-seeds pre-reduced by RSF under pre-exposure for (a) 2 h, (b) 4 h, (c) 8 h, and (d) 16 h. Scale bars, 100 nm.

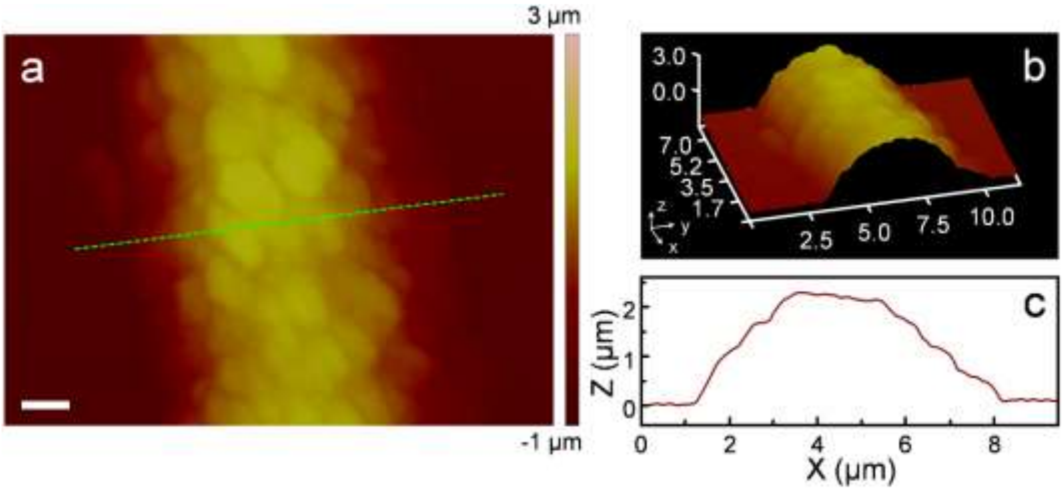

Supplementary Figure 8 | AFM characterization of the silk/Ag microwire connecting the two ITO electrodes in Figure 3g of main text. (**a**) 2D AFM graph of part of the silk/Ag microwire. Scale bar, 1 µm. (**b**) 3D-topography AFM graph of the silk/Ag microwire. Unit of coordinates, µm. (**c**) The cross-section profile of the silk/Ag microwire at the position of the green dotted line in (a).

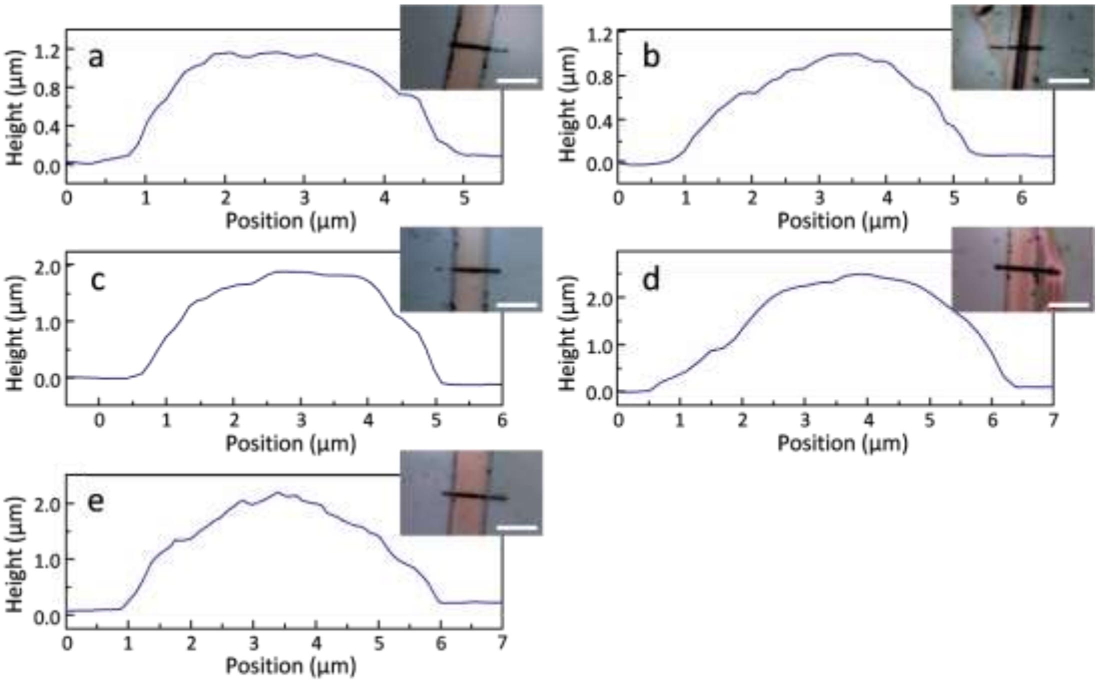

Supplementary Figure 9 | Cross-section profiles of silk/Ag micro-wires FsLDW-fabricated from inks with 3-wt% SF and different $AgNO_3$ concentrations ((**a**) 40; (**b**) 80; (**c**) 120; (**d**) 160; (**e**) 200; unit, mg·mL^{-1})**.** Insets, optical microscopic (OM) images of silk/Ag microwires; scale bars, 50 µm.

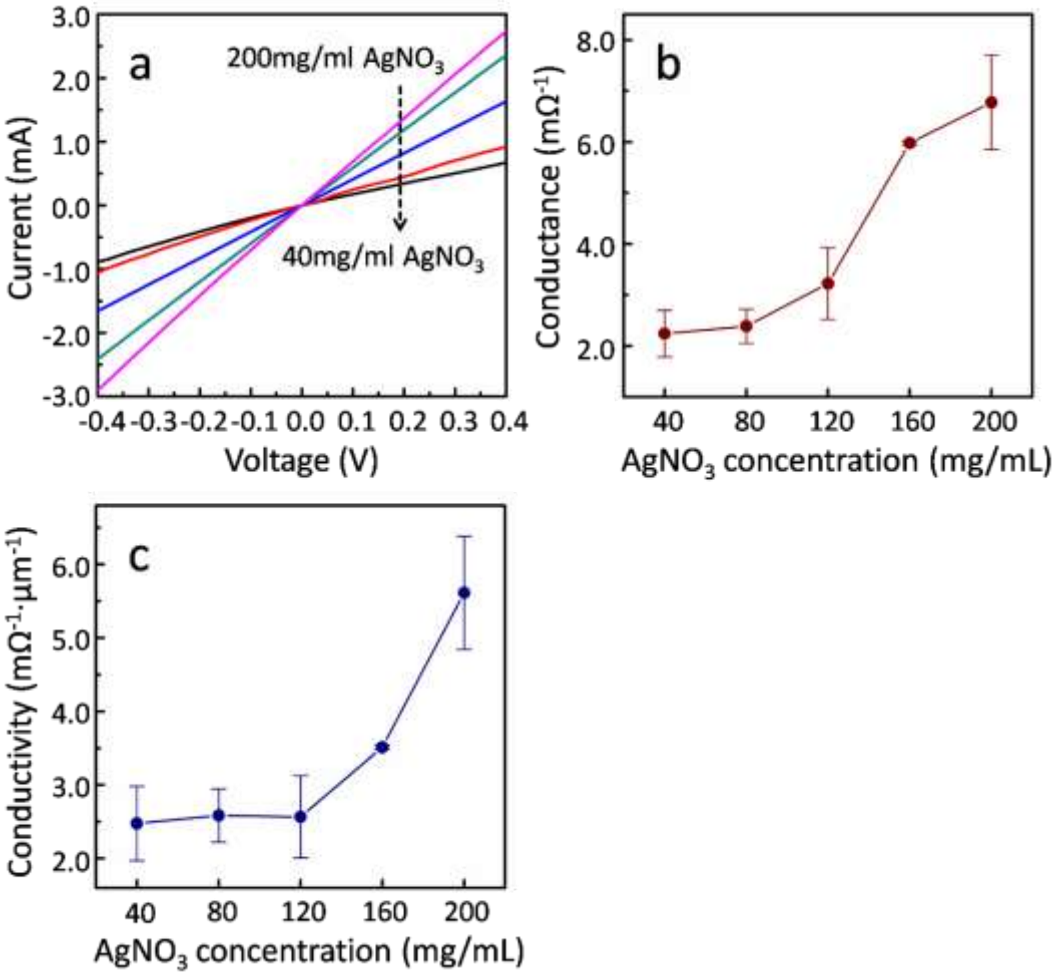

Supplementary Figure 10 | (**a**) Current-vs-voltage curves of silk/Ag micro-wires in Figure 1 of main text from inks with 3-wt% SF and different $AgNO_3$ concentrations (40-200 mg·mL^{-1}). (**b**) Electric conductance of silk/Ag micro-wires from inks with different $AgNO_3$ concentrations (40-200 mg·mL^{-1}). (**c**) Electrical conductivity of silk/Ag micro-wires from inks with different $AgNO_3$ concentrations (40-200 mg·mL^{-1}).

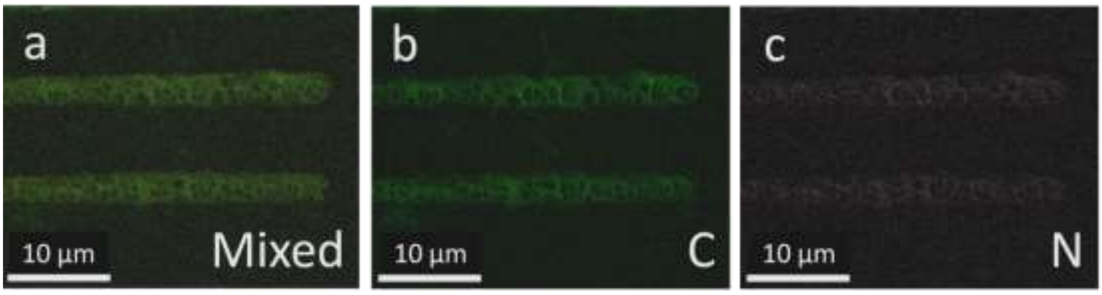

Supplementary Figure 11 | Mixed (a), elemental C (b) and N (c) EDS distribution maps of of silk/Au composite microwires in Figure 4a of main text. Scale bar, 10 μm.

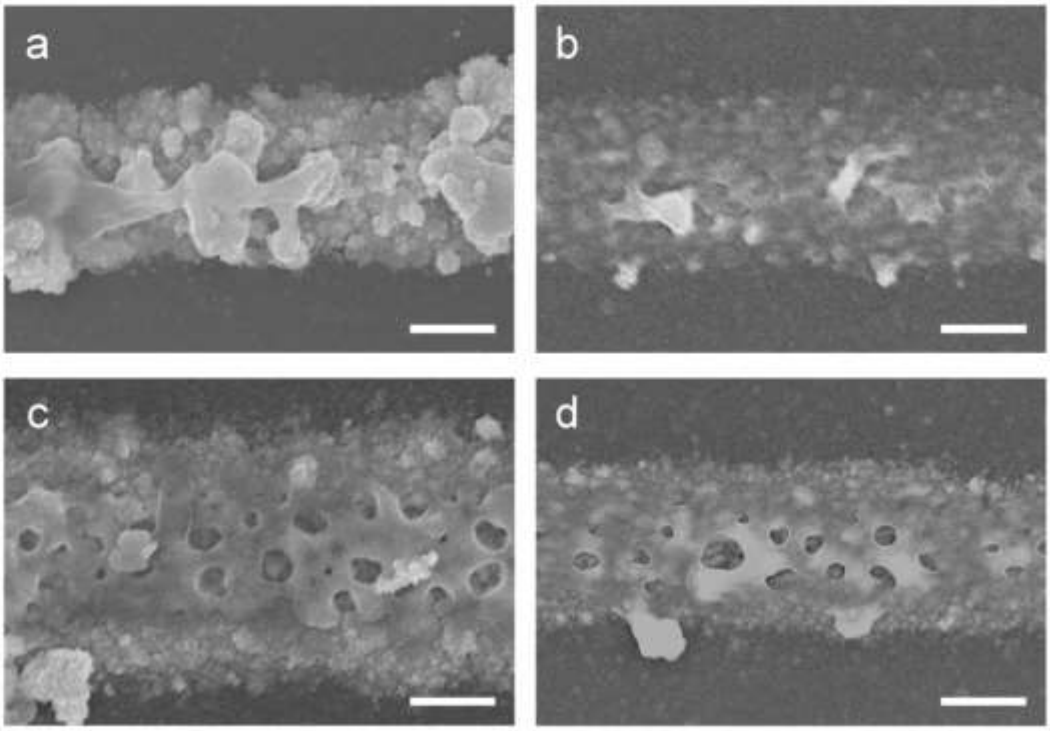

Supplementary Figure 12 | SEM images of silk/Au composite microwires FsLDW-fabricated from RSF/$HAuCl_4$ inks with (a) pH 1.0, (b) pH 3.0, (c) pH 5.0, and (d) pH 7.0. Scale bars, 1 μm.

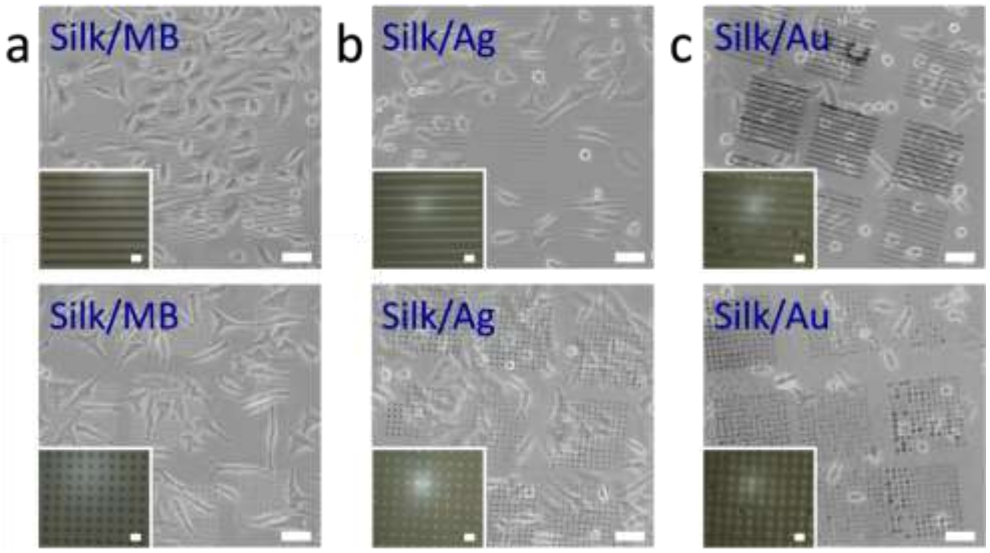

Supplementary Figure 13 | Optical microscopic (OM) images of L929 cells attached and incubated on silk-centered micropatterns; scale bars, 50 μm. Insets, metallographic microscopic images of micropattens without cells attached; scale bars, 10 μm. (**a**) L929 cells after 1-day incubation on pure silk based microwire arrays (top) and microdot arrays (bottom). (**b**) L929 cells after 1-day incubation on silk/Ag microwire arrays (top) and microdot arrays (bottom). (**c**) L929 cells after 1-day incubation on silk/Au microwire arrays (top) and microdot arrays (bottom).

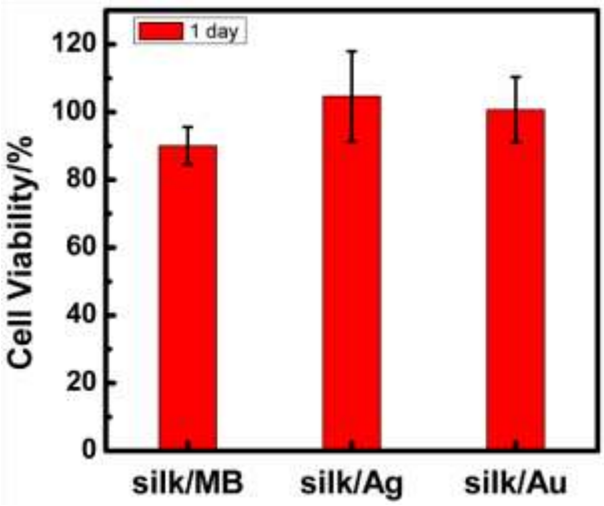

Supplementary Figure 14 | L929 cell viability after 1-day incubation on silk-centered micropatterns.

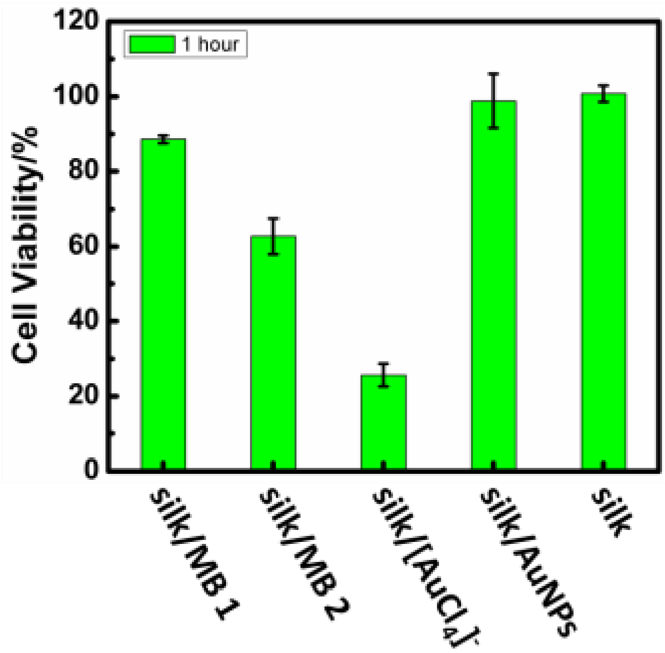

Supplementary Figure 15 | *Hela* cell viability after 1-hour incubation in silk-centered inks. All inks contain 0.37-wt% regenerated silk fibroin. Silk/MB 1, 0.17-mg·mL^{-1} MB; silk/MB 2, 0.34-mg·mL^{-1} MB; silk/$[AuCl_4]^-$ ink, 0.33-mg·mL^{-1} $HAuCl_4$, pH=7.0; silk/AuNPs ink was obtained by pre-exposuring above silk/$[AuCl_4]^-$ ink for 6 hours.

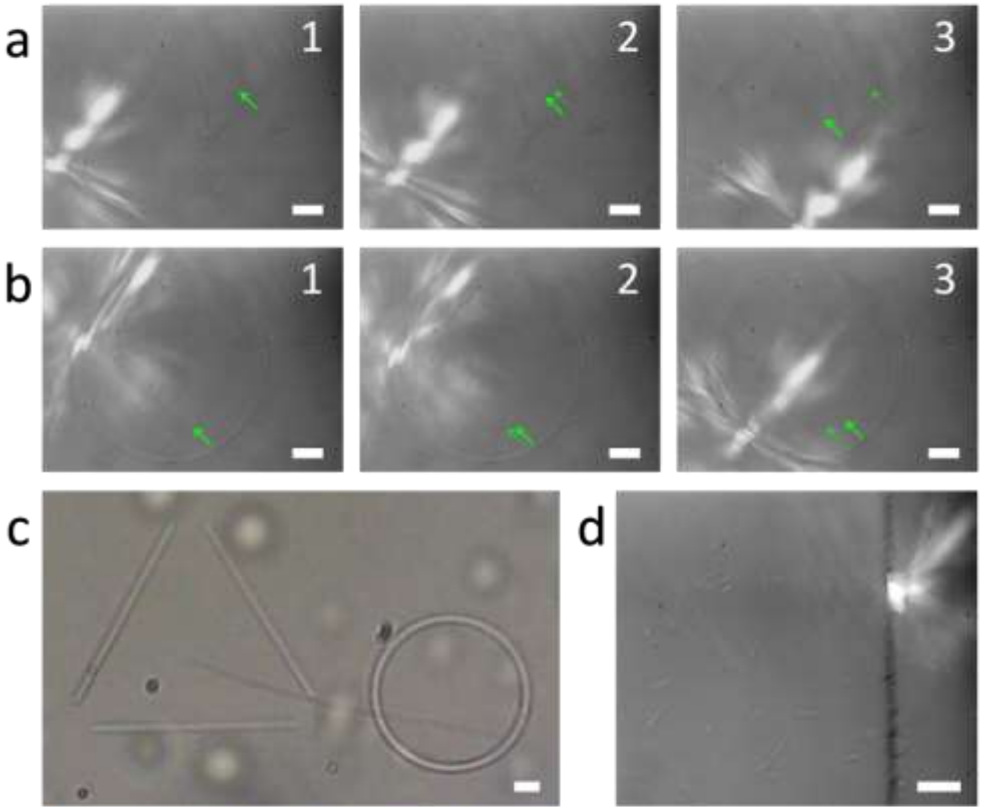

Supplementary Figure 16 | "In-situ" FsLDW when live bacteria (*Escherichia Coli* BL21) existed in the laser-processing areas; scale bars, 10 μm. (**a**) Snapshots from Movie 1 of FsLDW fabricating a micro-triangle in silk/MB ink containing live bacteria. (**b**) Snapshots from Movie 2 of FsLDW fabricating a micro-circle in silk/MB ink containing live bacteria. Green arrows in (a) and (b) pointed at the bacteria swimming across the laser-processing areas; the dashed ones marked the original positions in the 1st images. (**c**) Silk-based micropatterns obtained after FsLDW presented in (a) and (b) and water rinsing. (**d**) A snapshot from Movie 3 of FsLDW fabricating a micro-wire in silk/$[AuCl_4]^-$ ink containing live bacteria.

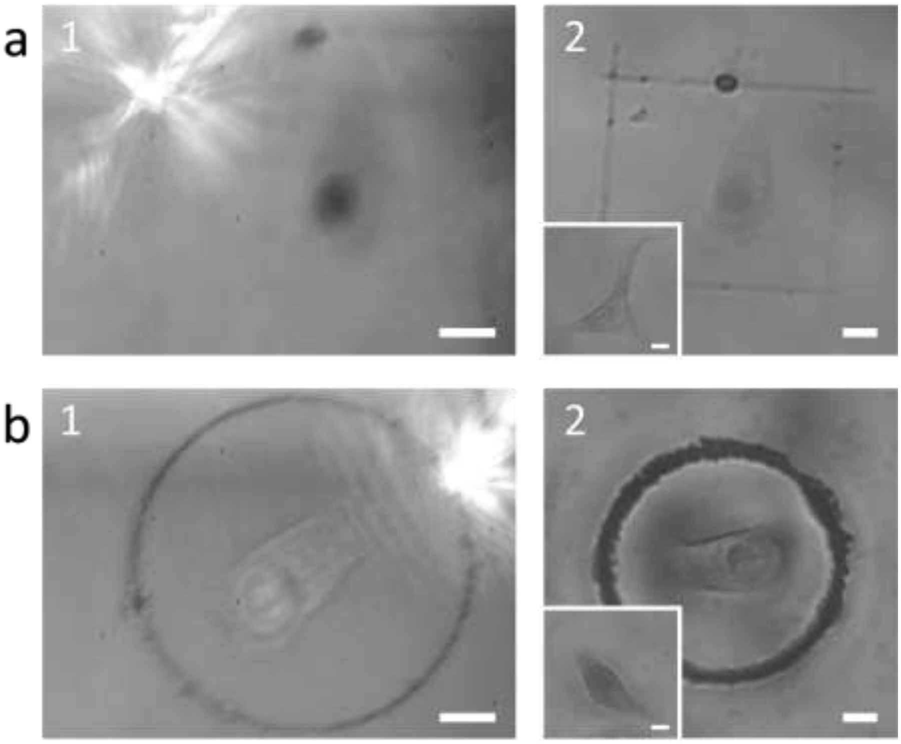

Supplementary Figure 17 | "In-situ"-FsLDW right near or around live *Hela* cells already attached on the glass slices; scale bars, 10 µm. (**a**) **1,** a snapshot from Movie 4 of FsLDW fabricating a micro-square in silk/MB ink. **2,** Obtained silk-based micro-square around the Hela cell; inset, the *Hela* cell a little far from laser-processing area. (**b**) **1,** a snapshot from Movie 5 of FsLDW fabricating a micro-circle in silk/$[AuCl_4]^-$ ink. **2,** Obtained silk/Au micro-circle around the *Hela* cell; inset, the *Hela* cell a little far from laser-processing area.

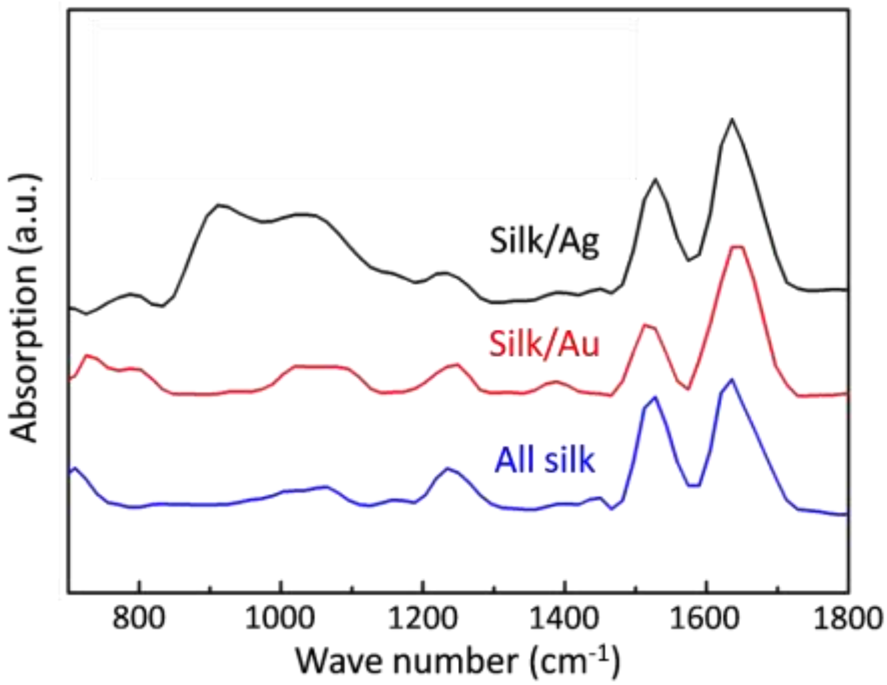

Supplementary Figure 18 | Attenuated-total-reflection Fourier transform infrared (ATR-FTIR) spectra of FsLDW-fabricated silk/Ag (black), silk/Au (red) and all-silk (blue) micro-patterns.

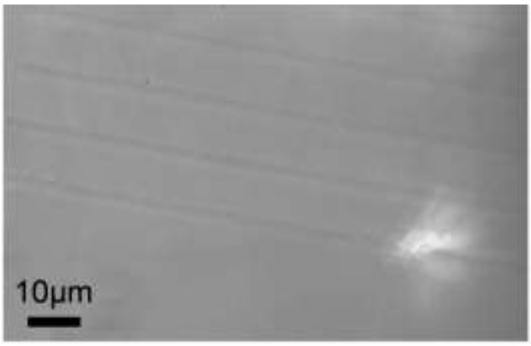

Supplementary Figure 19 | FsLDW in silk/MB ink with Fs-laser power intensity of ~ 75 mW·μm^{-2} (snapshot from Movie 6). Exposure time on single point, 1000 μs; scanning steps, 100 nm; NA=1.35.

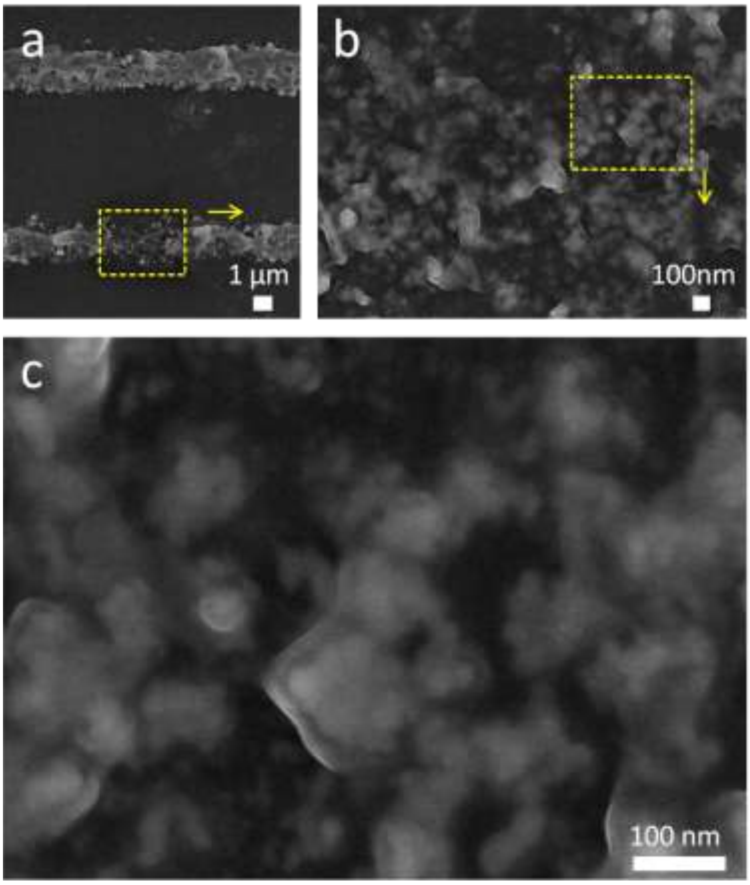

Supplementary Figure 20 | (**a**) FsLDW-fabricated silk/Au microwires with an occasionally broken breach (marked by the dashed box) exhibiting inside structure (scanning electron microscopic (SEM) image). Scale bar, 1 μm. (**b**) Enlarged SEM image of the marked area in (a). Scale bar, 100 nm. (**c**) Further enlarged SEM image of the marked area in (b). Scale bar, 100 nm.

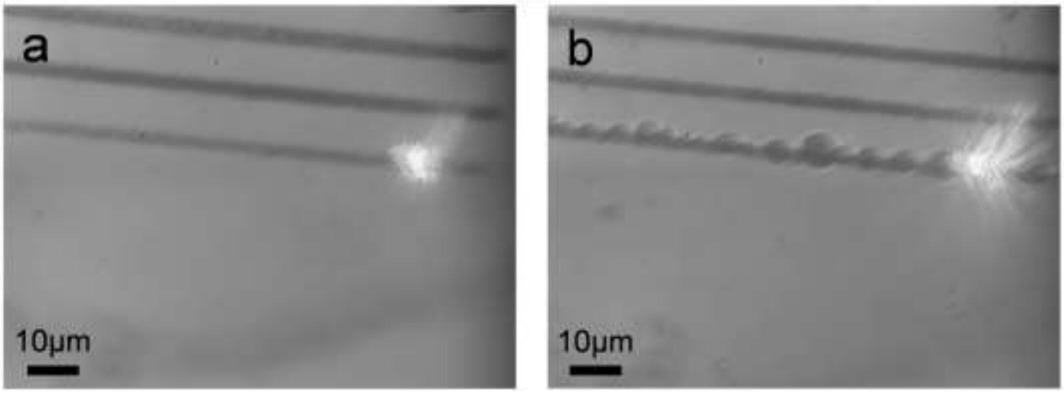

Supplementary Figure 21 | FsLDW in silk/$[AuCl_4]^-$ ink with Fs-laser power intensities of (**a**) ~ 2.1 mW·μm^{-2} (snapshot from Movie 7) and (**b**) ~ 3.5 mW·μm^{-2} (snapshot from Movie 8). Exposure time on single point, 1000 μs; scanning steps, 100 nm; NA=1.35. The laser powers were measured after the objective lens and glass-slice substrate. Scale bars, 10 μm.

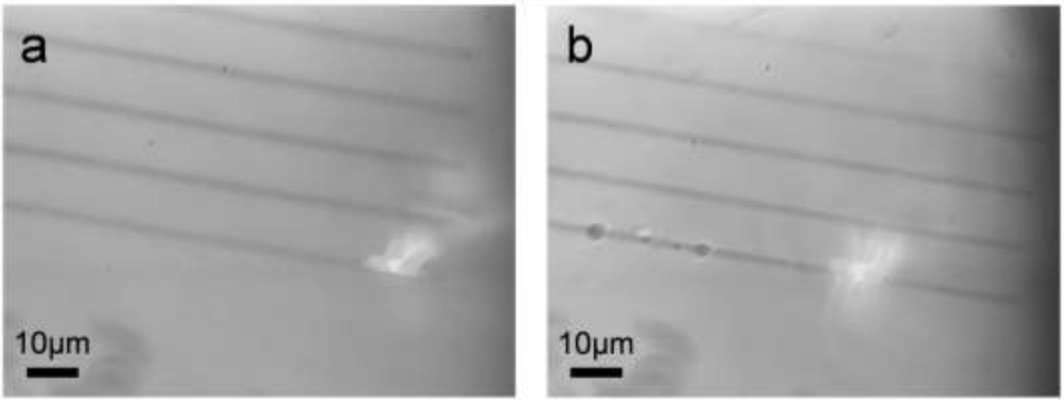

Supplementary Figure 22 | FsLDW in silk/$AgNO_3$ ink with Fs-laser power intensities of (**a**) ~ 3.5 mW·μm^{-2} (snapshot from Movie 9) and (**b**) ~ 5 mW·μm^{-2} (snapshot from Movie 10). Exposure time on single point, 1000 μs; scanning steps, 100 nm; NA=1.35. The laser powers were measured after the objective lens and glass-slice substrate. Scale bars, 10 μm.

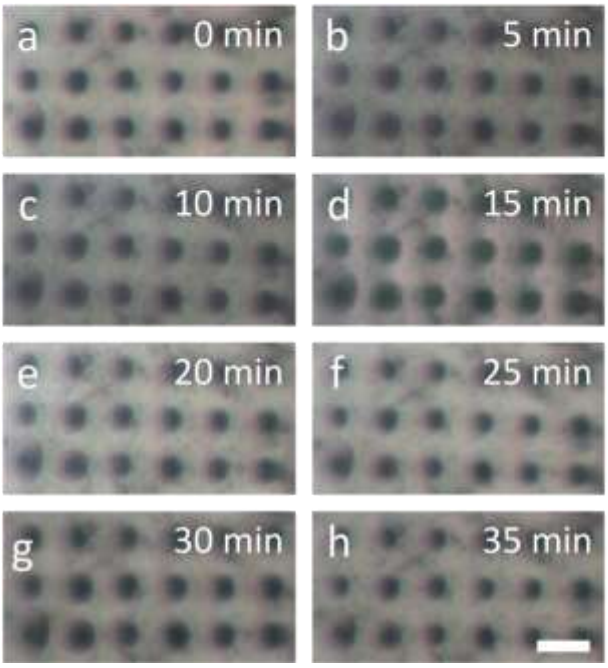

Supplementary Figure 23 | SF microdots FsLDW-fabricated from silk/MB ink were immersed in 0.83-g·mL^{-1} LiBr aqueous solution (60 °C) for up to 35 minutes. (**a**) - (**h**), metallographic optical microscopic images of the SF microdots after immersion for different time. Scale bar, 10μm.

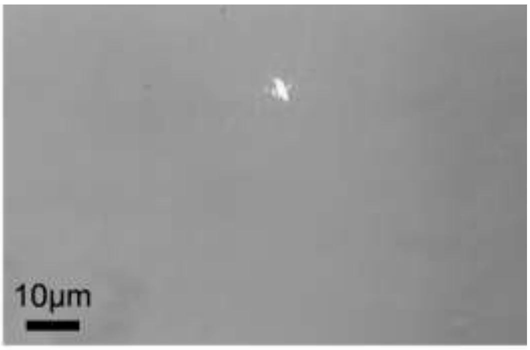

Supplementary Figure 24 | No curing was observed when Fs-laser was focused in pure SF aqueous solution for seconds (~ 75 mW·μm^{-2}, snapshot from Movie 11). The laser powers were measured before the objective lens (~ 27 mW·μm^{-2} after the objective lens) and glass-slice substrate. Scale bars, 10 μm.

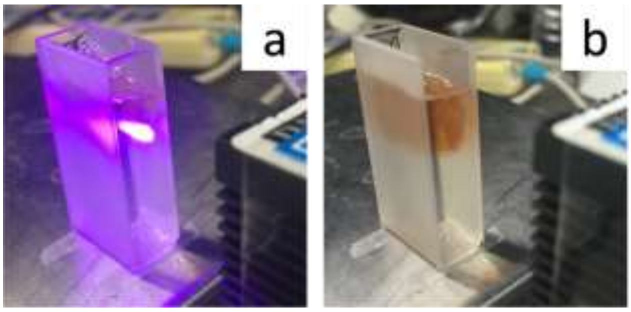

Supplementary Figure 25 | The fresh silk/$AgNO_3$ ink in the quartz cuvette was irradiated by 405-nm laser light. (**a**) the 405-nm laser is on. (**b**) AgNPs formed and obvious brown color appeared after only seconds' 405-nm exposure.

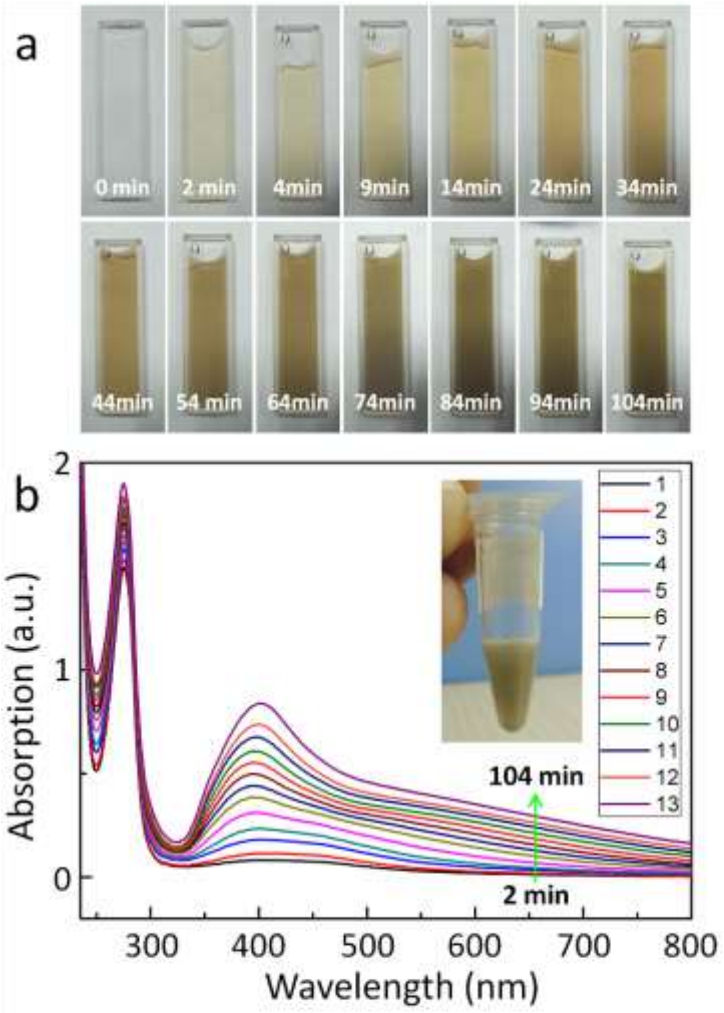

Supplementary Figure 26 | (**a**) Silk/$AgNO_3$ inks with increased exposure time under 405-nm laser light. (**b**) Ultraviolet-visible spectra of silk/$AgNO_3$ inks with different 405-nm-exposure time; the curves from 1 to 13 were obtained respectively from samples in (a) with exposure time from 2 to 104 minutes; inset, the silk/$AgNO_3$ ink after 104-minute 405-nm exposure.

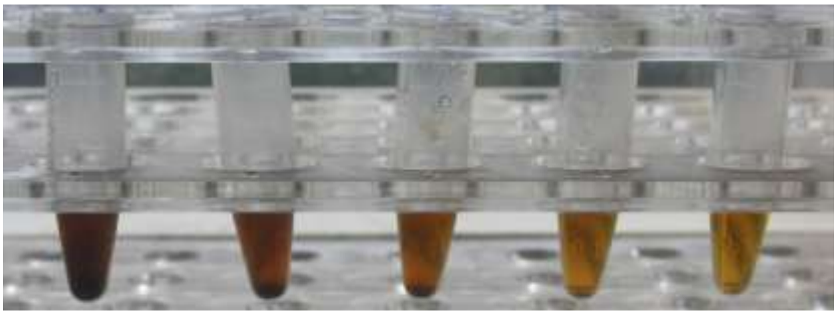

Supplementary Figure 27 | Images of silk/$AgNO_3$ inks 405-nm-exposed for different time (from right to left: 2, 9, 24, 44, 74 minutes)**.**

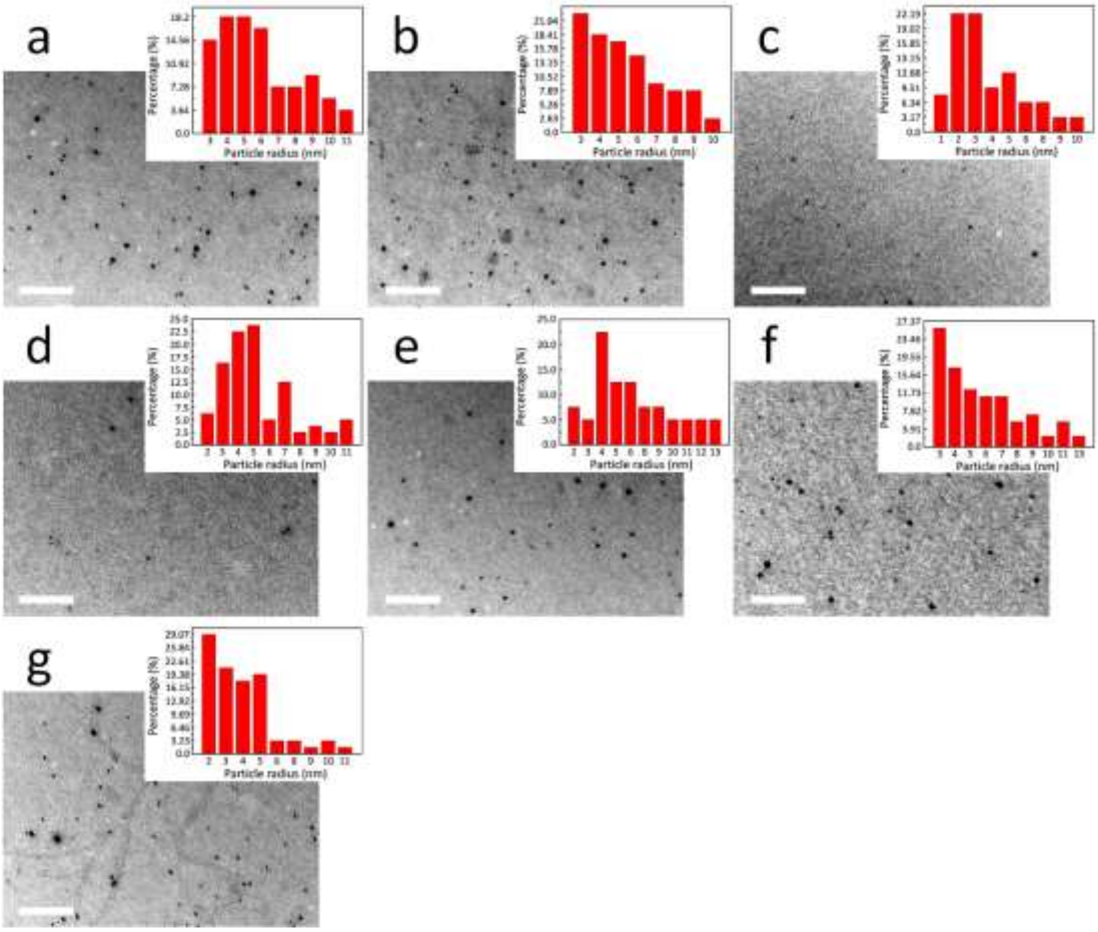

Supplementary Figure 28 | Transmission electron microscopic (TEM) images of air-dried films form silk/$AgNO_3$ inks after 405-nm exposure for (**a**) 30 min, (**b**) 35 min, (**c**) 40 min, (**d**) 45 min, (**e**) 50 min, (**f**) 55 min, (**g**) 60 min. Insets, radius distributions of AgNPs in silk/$AgNO_3$ inks (obtained by directly measuring in the TEM images). Scale bars, 200 nm.

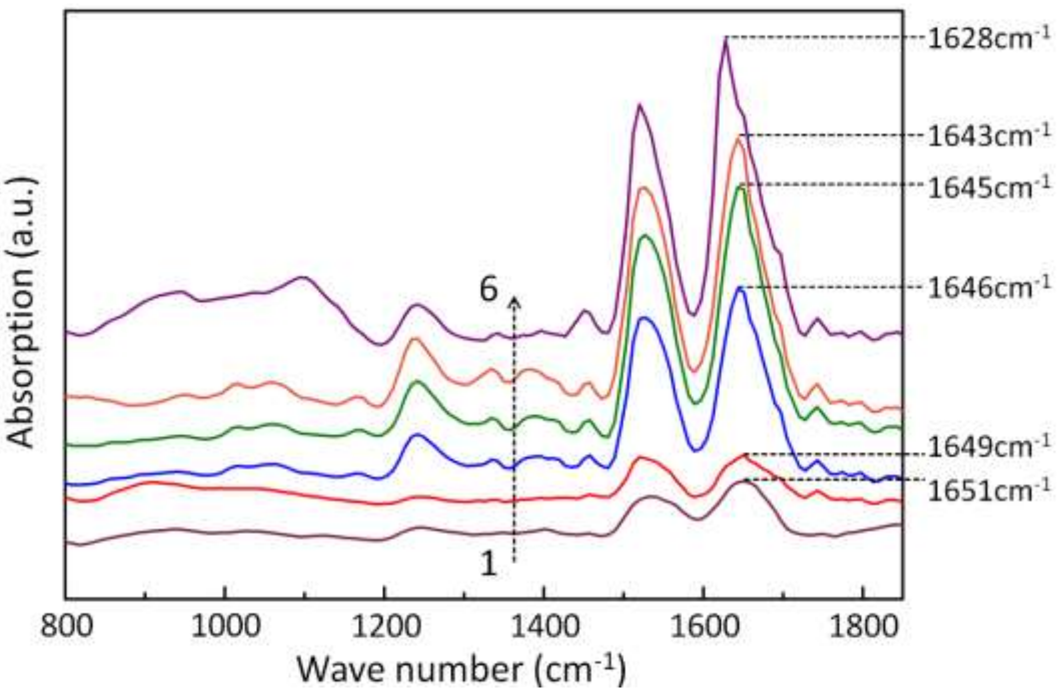

Supplementary Figure 29 | Attenuated-total-reflection Fourier transform infrared spectra (ATR-FTIR) of films obtained by spinning silk/$AgNO_3$ inks with different 405-nm exposure time. Curve 1, 0 min; 2, 4 min; 3, 24 min; 4, 44 min; 5, 64 min; 6, 94 min.

Supplementary Methods

Some experimental details of silk-centered FsLDW. The fabricated structures were directly left and supported on FsLDW substrates (glass or ITO slices as needed) after water-rinsing. Before using, SF-centered inks were kept under 4 °C and in the dark for preventing self-gelling of SF and photo-accelerated reduction of metal ions. For pre-exposure, SF/$AgNO_3$ or SF/$[AuCl_4]^-$ inks were sealed in polystyrene-based centrifugal tubes, and exposed under an incandescent light bulb (40 W) for appropriate time. During FsLDW, two details needed to be paid attention: i) proper encapsulation of FsLDW-fabrication area for restraining ink evaporation; ii) avoiding long-time exposure of high-intensity illumination (a fiber optic light is used for illumination and observation during FsLDW in our experiment). After FsLDW, the SF-centered micro/nano-structures were easily developed via water-rinsing and obtained on substrates. As-formed SF-centered micro/nano-structures/devices can keep their stable appearance during long-time storage in proper environments, for example, in dry air or pure water in our work.

Adjustment of electric conductivity of silk/Ag microwires (Supplementary Figure 9 and Supplementary Figure 10). We investigated the adjustment of silk/Ag microwire electro-conductivity by changing $AgNO_3$ concentration in inks. Along with higher $AgNO_3$ concentration in inks, micro-silk/Ag exhibited higher electric conductivity (see Supplementary Figure 9 and Supplementary Figure 10). In Supplementary Figure 10, the electric conductivity (K) was calculated by the formula as follow,

$$K = G \cdot L/S \tag{1}$$

where G was electric conductance (G=current/voltage), L was the length and S was the cross-section area of a silk/Ag micro-wire.

Supplementary Discussion

Analysis of indentation loading curves (Supplementary Figure 4). In Figure 2e of main text and Supplementary Figure 4c, the indentation loading curves were presented for a FsLDW-fabricated all-SF-based micro-square (20×20×4 μm^3) under dry condition in air (red curve, about 25% relative humidity, 25 °C) and equilibrated in water (blue curve), respectively. As the indicative of the stiffness, the curve slope of contact parts (vertical displacement of Z-piezo > 0) was obviously smaller for the RSF based micro-square immersed in water (red curve) than that dried in air (blue curve). This might be caused by that the RSF based micro-square was significantly equilibrium-swollen and therefore softened in water. Along with continuous AFM indentation, RSF-based biopolymers swollen in water might be compacted, and exhibited increasing indentation-loading curve slope and stiffness consequently (see blue curve in Figure 2e of main text and Supplementary Figure 4c). So it indicated that FsLDW-polymerized RSF-based biopolymers here acted like crosslinking hydrogel in aqueous environments with water absorption and extrusion. Moreover, in air with about 25% relative humidity, water layer existed on surface of both AFM tip and RSF based micro-square. When getting close to the RSF based micro-square, the AFM tip could be motivated by capillary forces to suddenly jump into contact with the RSF based micro-square, resulting into downward deflections in red curve of AFM indentation test in air (Figure 2e of main text and Supplementary Figure 4c)[32].

Silk/Ag composite microdevices for bio-electrics/electronics (Supplementary Figure 8). Relatively high Ag^{+} concentration (150-mg·mL^{-1} $AgNO_3$ in 1.5-wt% RSF aqueous solution) was applied for applicable electric conductibility (see **Methods**). As displayed in Figure 3h of main text, current-voltage curves of the silk/Ag composite microwire in air (25-% relative humidity, 23 °C) were tested, resulting electrical resistances of about 118 Ω (intact ITO film and unconnected ITO electrodes as controls). By AFM characterization, 3D topography of the silk/Ag microwire was measured (**Supplementary Figure 8**), by which the cross sectional area was estimated to be about 10 μm^2 (length, 100 μm; width, 7 μm; thickness, 2 μm). According to Ohm's law, the electric conductivity of the silk/Ag microwire was calculated to be about 8.47×10^4 $\Omega^{-1}\cdot m^{-1}$, which was about 3 order of magnitude smaller than that of bulk silver (6.25×10^7 $\Omega^{-1}\cdot m^{-1}$).

Details of silk-centered FsLDWs for bio-related applications (Supplementary Figure 13-17). Remarkably, by using silk/MB or silk/$[AuCl_4]^-$ inks with confirmed acceptable biocompatibility (**Supplementary Figure 15**), we demonstrated the feasibility of "in-situ" aqueous FsLDW where live bacteria (**Supplementary Figure 16**, **Movie 1**, **Movie 2** and **Movie 3**) or cells (**Supplementary Figure 17**, **Movie 4** and **Movie 5**) were right close to the laser-processing micro-areas. Based on its inherent high precision, low collateral thermal damage, noncontact feature, flexibility and facile designability, Fs-laser processing wouldn't induce significant impact on live bacteria or cells nearby. This may have great potential for "live" (real-time) and "in-situ" micro/nano-level cell manipulation, which is fairly difficult to implement via other nano-precision photo-processing.

In the experiments, human cervical cells (*Hela* cells) and mice fibroblast cell line (L929) were cultured in Dulbecco's modified Eagle's medium (DMEM) supplemented with 10% heat-inactivated fetal bovine serum (FBS) and antibiotics (100 $\mu g \cdot mL^{-1}$ streptomycin and 100 $\mu g \cdot mL^{-1}$ penicillin). Both cell lines were cultured at 37 ^oC in the humidified atmosphere with 5% CO_2. Cell Counting Kit-8 (CCK-8) WST-8
2-(2-methoxy-4-nitro-phenyl)-3-(4-nitrophenyl)-5-(2,4-disulfophenyl)-2H-tetrazolium monosodium salt) assay was employed for assessment of cytotoxicity in our experiment. CCK-8 was purchased from DojindoLaboratorise, Japan.

First, we used the FsLDW-fabricated silk-centered micropatterns (arrays) as substrates to attach and incubate cells (L929 cells for demonstration). Glass slices with SF micropatterns were place in the 12-well cell-culture plate after 1h UV sterilization. L929 cells dispersed in cell-culture plate (1ml in each well containing 1.0×10^5 cells per well) were cultured at 37 ^oC for 1day under 5% CO_2. To ensure reproducibility, three independent experiments were performed and all measurements were carried out in triplicate. Then, the culture medium was replaced with fresh serum-free DMEM, and CCK-8 was added in dark according to the protocol and the absorbance of the solution at 450nm was tested by and Elx 800

instrument (Biotek, USA) after further incubation for 2h. The relative viability was calculated as:

$$\text{Cell viability (\%)} = ([A]\text{test}-[A]\text{ blank}) / ([A]\text{control}-[A]\text{blank}) \times 100\% \qquad (2)$$

Where [A]test is the absorbance of the test sample, [A]blank is the absorbance of the blank sample without sample or cells and [A]control is the absorbance of the control sample without materials.

All FsLDW-fabricated silk-centered microstructures show a fairly good biocompatibility. As shown in **Supplementary Figure 13**, the L929 cells maintained good morphology after 1-day incubation. Cell viability was high for all silk-centered microwire or microdot arrays (pure silk micropatterns with a little MB remained, ~ 90% cell viability; silk/metal-Ag micropatterns, ~ 105%; silk/metal-Au micropatterns, ~ 101%; see **Supplementary Figure 14**).

Besides, we also experimentally tested the toxicity of silk-centered aqueous inks as recommended (Supplementary Figure 15). As mentioned by the reviewer, we observed that live cells would die in a short time (several minutes, confirmed by cell morphology) in inks containing high-concentration Ag^+ or AgNPs. However, for silk/MB, silk/$[AuCl_4]^-$ or silk/AuNPs inks, biocompatibility was acceptable. As FsLDW-fabrication of micro/nanostructures here usually took a short time from a few seconds to tens of minutes, we investigated cell viability after 1-hour incubation in silk-centered inks (see Supplementary Figure 15). After 1-hour incubation, cell viability ~89% and ~63% in silk inks containing 0.17-mg·mL^{-1} and 0.34-mg·mL^{-1} MB, respectively. By pre-exposuring silk/$[AuCl_4]^-$ ink (~ 6 hour under Incandescent light), part of $[AuCl_4]^-$ ions were reduced into AuNPs and cell viabilitywas also largely improved from ~26% to ~99% cell viability after 1-hour incubation.

In the experiments, *Hela* cells were seeded in 96-well plates (100μL in each well containing 1.0×10^4 cells per well) with culture medium and incubated at 37 ^oC for 24h under 5% CO_2. After removing the medium, 200μL of DMEM with different concentration of work solution were added and incubated for 1h. To ensure

reproducibility, three independent experiments were performed and all measurements were carried out in triplicate. Then, the culture medium was replaced with fresh serum-free DMEM, and CCK-8 was added in dark according to the protocol and the absorbance of the solution at 450nm was tested by and Elx 800 instrument (Biotek, USA) after further incubation for 2h. The relative viability was calculated as:

$$\text{Cell viability (\%)} = ([A]\text{test}-[A]\text{ blank}) / ([A]\text{control}-[A]\text{blank}) \times 100\% \qquad (3)$$

Where [A]test is the absorbance of the test sample, [A]blank is the absorbance of the blank sample without sample or cells and [A]control is the absorbance of the control sample without materials.

Based on the above results, we demonstrated the feasibility of "in-situ" aqueous FsLDW where live bacteria (**Supplementary Figure 16**, **Movie 1**, **Movie 2** and **Movie 3**) or cells (**Supplementary Figure 17**, **Movie 4** and **Movie 5**) were right close to the laser-processing micro-areas. In detail, fine silk-based microstructures were FsLDW-fabricated from silk/MB ink containing live *Escherichia Coli* BL21 (**Supplementary Figure 16**, **Movie 1** and **Movie 2**). Although several bacteria were occasionally caught by scanned laser, most of the *Escherichia Coli* BL21 kept alive and were not impacted obviously by the nearby laser processing (see **Movie 1** and **Movie 2**). Live bacteria could be even observed to be swimming directly across the laser fabrication domains (as marked by green arrows in Supplementary Figure 16 (a) and (b)). The pure-silk-based micropatterns were finally obtained after water-rinsing and shown in Supplementary Figure 16 (c). Besides, silk/Au microstructures were also Fs-laser written out from silk/$[AuCl_4]^-$ ink containing live *Escherichia Coli* BL21 (Supplementary Figure 16 (d)). On the experimental basis in Supplementary Figure 15, the silk/$[AuCl_4]^-$ ink was pre-exposured under incandescent light for 1 hour or stored in dark for 4-6 hours (4 ^{o}C) to partly reduce $[AuCl_4]^-$ ions into AuNPs and improve the ink biocompatibility. Further, we fabricated silk/Au microstructures via "in-situ"-FsLDW right near or around live *Hela* cells already attached on the glass slices. In **Supplementary Figure 17 (a)** and **Movie 4**, a micro-square was laser-written out around a *Hela* cell from silk/MB ink. Similarly, a micro-circle was fabricated around another *Hela* cell in silk/$[AuCl_4]^-$ ink (see **Supplementary Figure 17 (b)** and **Movie 5**). The *Hela* cells near or a little far from (insets in Supplementary

Figure 17) the laser-processing areas all exhibited normal morphology after and during the FsLDW.

It should be noted that all the silk-centered inks need to be tuned to have neutral pH value (around 7.0) and osmotic pressure (approximately equivalent to that of 0.9-wt% NaCl aqueous solution). The RSF mother solution was dialyzed in 0.9-wt% NaCl aqueous solution for 24 hours. Then, it was used to prepare all the inks (silk/MB, silk/$[AuCl_4]^-$, silk/AuNPs) related to ink cell-culturing and "in-situ" FsLDW. Meanwhile, 0.9-wt% NaCl aqueous solution was used as the solvent to prepare all the other solutions to obtain these FsLDW bio-inks. In detail, the compositions of the related silk-centered inks are as follow,

i) silk/MB ink: silk mother solution (100 μL) + 3-mg·mL^{-1} MB solution (20 μL, using 0.9-wt% NaCl solution as solvent);

ii) silk/$[AuCl_4]^-$ ink: silk mother solution (20 μL) + 0.9-wt% NaCl solution (90 μL) + 5-mg·mL^{-1} $HAuCl_4$ solution (90 μL, using 0.9-wt% NaCl solution as solvent); $HAuCl_4$ solution was pre-titrated to pH=7.0 using saturated NaOH solution (~ 1 g·mL^{-1}, such high concentration is adopted for little change of ion strength after titration));

iii) before "in-situ" FsLDW, silk/$[AuCl_4]^-$ ink was pre-exposured under incandescent light for 1 hour or stored in dark for 4-6 hours (4 ^{o}C) to partly reduce $[AuCl_4]^-$ ions into AuNPs and improve the ink biocompatibility.

Thus, as-prepared silk-centered inks have osmotic pressure approximately equivalent to that of normal saline (0.8-0.9 wt% NaCl aqueous solution), which is important for keep bacteria or cells alive in the ink during FsLDW. For "in-situ" FsLDW of *Escherichia Coli* BL21, the silk-centered inks were mixed with original suspension of bacteria (optical density, 1.0) at 4 :1 in volume. For "in-situ" FsLDW of *Hela* cells, DMEM suspension of *Hela* cells was added onto the glass slices. Then, the samples were incubated under 37 ^{o}C for about half an hour to make *Hela* cells attach on glass substrate. Subsequently, when only a liquid film was left after drawing the DMEM nutrient solution off, silk-centered inks were added at the areas for fabrication on glass slices. Then, the samples were sealed and positioned on the FsLDW stages

for laser processing followed.

Detailed data and discussion on probable focal temperature in silk-centered inks during FsLDW (Supplementary Figure 19-22). It has demonstrated that photothermal effect in water is very similar to that in ocular and other biological media (probably also including silk/MB ink used here) [1, 2]. According to the conclusion in Prof. G. Paltauf's work, the theoretical temperature increase tend to be stable at ~ 1.1 °C after Fs-laser exposure for more than 1 μs (800-nm and 100-fs pulses focused in water; 80-MHz repetition rate; objective NA, 1.3), when **deposited** volumetric energy density per pulse is 1 J·cm^{-3} at the focus center. And the final temperature varies proportionally to the volumetric energy density of Fs-laser. This situation is quite similar with our work (800-nm and 120-fs pulses focused in aqueous inks; 80-MHz repetition rate; objective NA, 1.35). As during FsLDW in our work, Fs-laser exposure at a single point is long enough to produce stable temperature distribution (1000 μs or longer, much longer than 1 μs).

First, **<u>for silk/MB ink</u>**, ~75-mW·μm^{-2} (measured **before** the objective lens) Fs-laser average power intensity is applied as the optimal FsLDW parameter. When the focused laser beam passed through the silk/MB ink, about 6.3-mW·μm^{-2} laser power would be deposited (experimentally measured value; equivalent to ~ 66 J·cm^{-3} per pulse; focal spot is an approximate ellipsoid with ~1800-nm major axis and ~700-nm minor axes). Accordingly, the focal temperature might be increased by ~ 73 °C (from ~ 22-°C room temperature to ~ 95 °C, without obvious boiling/cavitation) as the enough long Fs-laser irradiation (**Movie 6** and **Supplementary Figure 19**). Besides,

the smallest line width is ~ 350-400 nm when using Fs-laser intensity of ~ 75 mW·μm^{-2} in our work (see Figure 2(a) in the manuscript). It is precisely consistent with the span of 1000-μs isothermal profile at ~50-°C increase of temperature (namely, ~ 70-°C local temperature that could cause SF β-conformation transition [3]) in radial direction of laser focus [1]. This coincidence implies that the probable temperature distribution in laser focal spot should be approximate to the calculation.

In addition, obvious SF curing started at about 50-mW·μm^{-2} laser power intensity (measured before the objective lens). Accordingly, ~ 4.2-mW·μm^{-2} laser power (~ 44 J·cm^{-3} per pulse) was deposited and it induced ~ 70-°C focal temperature. Thus, focal temperature from **~ 70°C to ~ 95°C** could be resulted in during FsLDW in silk/MB aqueous ink.

As for silk/Ag^+ and silk/$[AuCl_4]^-$ inks, it becomes more complicated as the presence of nano-metals. Photothermal effect can be significantly enhanced due to plasma resonance effect [4, 5]. To achieve an optimal balance between energy absorption and heat diffusion, and therefore a maximum temperature increase, the optimum size of AuNPs should be around 40-50 nm [4]. For an ideal condition, a steady ~30-°C temperature increase will be resulted in water proportionally along with every 1.0-mW·μm^{-2} increase of average Fs-laser power intensity (AuNP diameter, 50-nm; wavelength, 800 nm; repetition frequency, 86 MHz) [4, 5]. Different from photo-heating calculation in water or silk/MB ink mentioned above, the two key parameters for photothermal effect of nano-metals are optical absorption cross section and laser-illumination optical power intensity, instead of the final light energy deposition.

The AuNPs made by SF in our (**Supplementary Figure 20**) and other work [6], are around 10-50 nm. **For silk/$[AuCl_4]^-$ ink**, slight micro/nano-curing could be observed under Fs-laser intensity as low as ~ 0.75 mW·μm^{-2} (measured **after** glass slice; exposure time on single point, 1000 μs; scanning step, 100nm; NA=1.35). The resulted maximum increase of temperature could be ~ 22.5 °C for 50 nm AuNPs. By increasing laser power to ~ 2.1 mW·μm^{-2}, a maximum focal temperature might be ~ **85 °C**, resulting in well curing SF (β-folding contained) with no boiling/cavitation

(see **Movie 7** and **Supplementary Figure 21a**). As the laser power intensity further increase to ~ 3.5 mW·μm^{-2} (about 12.5 mW·μm^{-2} measured before objective), the maximum focal temperature was theoretically calculated to be **~ 127 °C** (**the actual value should be obviously lower**). As a result, bubbling appeared occasionally (see **Movie 8** and **Supplementary Figure 21b**).

<u>For silk/$AgNO_3$ ink</u>, 10-20 nm AgNPs were produced with different pre-exposure time (**Supplementary Figure 6** and **Supplementary Figure 7** in supplementary information (SI)) [7]. In the experiments, higher Fs-laser power intensity could be used for silk/Ag^+ ink with no bubble observed in **Movie 9** and **Supplementary Figure 22a** (~ 3.5 mW·μm^{-2} **after** the objective lens and glass-slice substrate). Bubbles started to form as the Fs-laser intensity increased to over ~ 5 mW·μm^{-2}, (probably about 100 °C; also without obvious damage to fabricated microstructures; see **Movie 10** and **Supplementary Figure 22b**). Therefore, since focal temprature probably increased ~ 78 °C for 5-mW·μm^{-2} laser, 3.5-mW·μm^{-2} optimal power intensity should proportionally result into a 55-°C increase (about 77-°C focal temperature) [4, 5].

Details on probable mechanisms of silk-centered FsLDW (Supplementary Figure 23-29). We tested attenuated-total-reflection Fourier transform infrared (ATR-FTIR) absorption spectra of FsLDW-fabricated all-silk, silk/Ag, and silk/Au micro-patterns (see **Supplementary Figure 18**). First, the 1050-1150 cm^{-1} absorption strongly demonstrated formation of C-O-C bonds (probable result of Tyr oxidization and polymerization [8]), suggesting the oxidation crosslinking of RSF during FsLDW. As we mentioned, it has been widely accepted and applied to various proteins' FsLDW that enhanced production of singlet oxygen via two-photon MB-photosensitization ($^1O_2$, or other oxidizing species by other photosensitizers) promoted protein crosslinking under high-intensity irradiation of femtosecond laser pulses [9, 10]. Meanwhile, series of existing researches have also experimentally demonstrated that light-irradiated nano-metals did enhance localized electromagnetic field [11, 12, 13, 14], photo-thermal effects (also proved in our experiments described above), and photo-production of $^1O_2$ (or other oxidizing species) [15]. Therefore, we believe that the photo-oxidation crosslinking in our RSF system was probably also "catalyzed" by MB or nano-metals. Meanwhile, the amide-I absorption peak at 1635 cm^{-1} (shifted from 1651 cm^{-1} of amorphous SF) proved β-sheets in SF molecules likely formed by photo-heating during FsLDW [16]. Thus, these two simultaneous effects (oxidation-crosslinking and β-folding) illustrate why micro/nano-gels could be formed during FsLDW.

More detailed discussions are in the following.

In silk/MB ink, focal temperature could be around 70-95 °C during FsLDW with optimized processing parameters. Therefore, photoheating caninduce β-crystallization and cure SF from ink. However, "any thermal denaturation of biomolecules will thus always be mixed with free-electron-induced chemical effects, and the latter will probably dominate" [1]. Another evidence in our work is that FsLDW-fabricated SF microstructures could not be totally hydrolyzed and dissolved in dense LiBr aqueous solution (0.83 $g·mL^{-1}$, 60 °C; see **Supplementary Figure 23**). As we know, dense LiBr solution can efficiently dissolve β-conformation based SF microstructure(even raw silk fibers) by breaking hydrogen bonds and dissociating β-sheets [17]. Moreover,

Fs-laser-induced curing was not observed in pure SF aqueous solution (without MB or metal ions; **Movie 11** and **Supplementary Figure 24**). Therefore, these results further demonstrate that heating was not the main reason of curing SF. SF covalent crosslinking did occur under high-intensity Fs-laser as demonstrated by ATR-FTIR in Supplementary Figure 18.

For the silk/Ag^+ and silk/$[AuCl_4]^-$ inks, optimized FsLDW parameters (weaker than those for silk/MB ink) induced focal temperatures probably around ~80-100 °C as discussed above. Similarly, photothermal effect played an important part in micro/nano-curing SF. As for other probable photochemical processes, we investigated the UV single-photon process (e.g. ~ 400 nm) in inks, which is a reasonable and frequently used parallel of infrared multiphoton process (e.g. 800 nm).

405-nm laser light (~ 6.2 $mW\cdot mm^{-2}$) was used to irradiate fresh silk/$AgNO_3$ ink (4-wt% SF and 5-$mg\cdot mL^{-1}$ $AgNO_3$). Under UV exposure, the irradiated area of ink immediately became brown (**Supplementary Figure 25**) and it went darker and more feculent (**Supplementary Figure 26**), as the increase of exposure time. Correspondingly, the ultraviolet-visible (UV-vis) absorption of inks became more significant across wider wavelength range (~ 350-700 nm), suggesting the of AgNPs. After standing in dark for hours, more sediment deposited in longer UV-exposed silk/$AgNO_3$ inks (**Supplementary Figure 27**). Considering that diameters of AgNPs were about 6-10 nm (**Supplementary Figure 28**), the higher turbidity was probably related to crosslinks and SF micro/nano-gels formed in inks [18]. On the other hand, the ATR-FTIR absorption peak shifted step by step from 1651 cm^{-1} to 1628 cm^{-1} (curve 1 to 6 in **Supplementary Figure 29**) demonstrated the formation of β-sheet during increasing exposure. Importantly, infrared absorption around 1050-1150 cm^{-1} increased gradually with longer 405-nm exposure time (consistent with results of FsLDW-fabricated micro-silks in Supplementary Figure 18), proving the C-O-C bond formation (a clue of Tyr redox polymerization) [8]. Therefore, both two effects (oxidation-crosslinking/polymerization and β-sheet formation) involved in and enabled SF micro/nano-curing during FsDLW.

Accordingly, the photoreduction of Ag^+ under 800-nm Fs-laser irradiation might

also exist, which was supposed to be induced by a MPA process [11-14]. Furthermore, metal nanoparticles were reported to greatly help to reduce more metal [11-13] and to MPA-polymerize surrounding pre-polymers or monomers [13, 14] via enhanced plasmonic field. Especially, the nanomaterials of noble metal, such as Ag, Au, Pt, were well demonstrated and applied as photosensitizers to MPA-generate $^1O_2$ (or other oxidizing species) for photodynamic therapy and photopolymerization [15].

Actually, specific crosslinking mechanisms of various protein FsLDW (including SF) are very complex with plenty of reaction pathway and many probable catalysis mechanism. This issue still needs more in-depth investigations by chemists and we will also try to do more research in this domain.

Discussion on photoluminescence of FsLDW-fabricated RSF microstructures. Actually, SF itself originally has the ability of photoluminescence, for instance, a 305-nm-stimulated fluorescent peak at ~ 340 nm [18]. The enhanced fluorescence and even new peaks emerged in 400~470-nm region (305-nm excitation). Correspondingly, 405-nm-excited FsLDW-fabricated micro-silks exhibited enhanced fluorescence in 500-nm region in our work. It suggested that new photoproducts were formed during UV processing [18]. For example, phenylalanine and tyrosine crosslinked via photooxidation in the irradiated SF solution [19]; photooxidation of methionine could change SF fluorescence [20]; radicals formed in SF chain might interact with oxygen to form peroxy radicals [21]; carbonyl groups formed as a result of the photooxidation of SF to α-keto-acids at glycine and alanine residues [22]. So, these new photoproducts and crosslinks might bring new chromophores to change absorption and fluorescence spectra of SF. Importantly, di-tyrosine cross-links were known to emit around 400-nm fluorescence under 305-nm excitation (accordingly, 500-nm fluorescence under 405-nm excitation), which was considered to be related to fluorescent changes of UV-processed RSF (as well as SF microstructures here multiphoton fabricated and irradiated by 800-nm Fs laser; see Figure 5b) [18]. Namely, the changed fluorescence is also a clue indicating probable Tyr-involved covalent crosslinking of SF during FsLDW.

Supplementary References

[1] Vogel, A., Noack, J., Huttman, G., & Paltauf, G. Mechanisms of femtosecond laser nanosurgery of cells and tissues. *Appl. Phys. B* **81**, 1015-1047 (2005).

[2]Vogel, A., Linz, N., Freidank, S., & Paltauf, G. Femtosecond-laser-induced nanocavitation in water: implications for optical breakdown threshold and cell surgery. *Physical Review Letters***100**, 038102 (2008).

[3] Li, X. G., Wu, L. Y., Huang, M. R., Shao, H. L., & Hu, X. C. Conformational transition and liquid crystalline state of regenerated silk fibroin in water. *Biopolymers* **89** (6), 497-505 (2008).

[4] Baffou, G., & Rigneault, H., Femtosecond-pulsed optical heating of gold nanoparticles. *Physical Review B* **84**, 035415 (2011)

[5] Ekici, O., Harrison, R. K., Durr, N. J., Eversole, D. S., Lee, M., & Ben-Yakar. A. Thermal analysis of gold nanorods heated with femtosecond laser pulses. *J. Phys. D: Appl. Phys.* **41**, 185501 (2008).

[6] Zhou, Y., Chen, W., Itoh, H., Naka, K., Ni, Q., Yamane, H., & Chujo, Y. Preparation of a novel core–shell nanostructured gold colloid-silk fibroin bioconjugate by the protein in situ redox technique at room temperature. *Chem. Commun.* **23**, 2518-2519 (2001).

[7] Fei, X., Jia, M. H., Du, X., Yang, Y.H., Zhang, R., Shao, Z. Z., Zhao, X., & Chen, X. Green synthesis of silk fibroin-silver nanoparticle composites with effective antibacterial and biofilm-disrupting properties. *Biomacromolecules***14**, 4483-4488 (2013)..

[8] Shen, H.R., Spikes, J.D., Smith, C.J., & Kopecek, J. Photodymanic crosslinking of proteins-V. Nature of the tyrosine-tyrosine bonds formed in the FMN-sensitized intermolecular cross-linking of N-acetyl-L-tyrosine. *Journal of Photochemistry and Photobiology A: Chemistry* **133**, 115-122 (2000).

[9] Tardivo, J. P., Del Giglio, A., de Oliveira,C. S.,Gabrielli, D. S., Junqueira, H. C., Tada, D. B.,Severino, D., Turchiello, R. d. F., & Baptista. M. S. Methylene blue in photodynamic therapy: From basic mechanisms to clinical applications. *Photodiagnosis and Photodynamic Therapy* **2**, 175-191 (2005).

[10] Shen, H. R., Spikes, J. D., Smith, C. J., & Kopecek, J. Photodynamic cross-linking of proteins IV. Nature of the His–His bond(s) formed in the rose bengal-photosensitized cross-linking ofN-benzoyl-L-histidine. *Journal of Photochemistry and Photobiology A: Chemistry* **130**, 1-6 (2000).

[11] Shukla, S., Furlani, E. P., Vidal, X., Swihart, M. T., & Prasad, P. N. Two-photon lithography of sub-wavelength metallic structures in a polymer matrix. *Adv. Mater.* **22**, 3695-3699 (2010).

[12] Shukla, S., Vidal, X., Furlani, E. P., Swihart, M. T., Kim, K. T., Yoon, Y. K., Urbas, A., & Prasad, P. N. Subwavelength direct laser patterning of conductive gold nanostructures by simultaneous photopolymerization and photoreduction. *ACS Nano* **5**, 1947-1957 (2011).

[13] Xu, B. B., Wang, L., Ma, Z. C., Zhang, R., Chen, Q. D., Lv, C., Han, B., Xiao, X. Z., Zhang, X. L., Zhang, Y. L., Ueno, K., Misawa, H., & Sun, H. B. Surface-plasmon-mediated programmable optical nanofabrication of an oriented silver nanoplate. *ACS Nano* **8**, 6682-6692 (2014).

[14] Ueno, K., Juodkazis, S., Shibuya, T., Yokota, Y., Mizeikis, V., Sasaki, K., & Misawa, H. Nanoparticle plasmon-assisted two-photon polymerization induced by incoherent excitation source. *J. am. chem. soc.***130**, 6928-6929 (2008).

[15] Jiang, C., Zhao, T., Yuan, P., Gao, N., Pan, Y., Guan, Z., Zhou, N., & Xu, Q. H. Two-photon induced photoluminescence and singlet oxygen generation from aggregated gold nanoparticles. *ACS Appl. Mater. Interfaces* **5**, 4972-4977 (2013).

[16] Kim,S., Marelli, B., Brenckle1, M. A., Mitropoulos, A. N., Gil, E. S., Tsioris, K., Tao, H., Kaplan, D. L., & Omenetto, F. G. All-water-based electron-beam lithography using silk as a resist. *Nature Nanotechnology* **9**, 306-310 (2014).

[17] Wang, Q., Chen, Q., Yang, Y. H., & Shao, Z. Z. Effect of various dissolution systems on the molecular weight of regenerated silk fibroin. *Biomacromolecules* **14**, 285-289 (2013).

[18] Sionkowska, A., & Planecka, A. The influence of UV radiation on silk fibroin. *Polymer Degradation and Stability* **96**, 523-528 (2011).

[19] Sionkowska A, Kaminska A, Miles C. A., &Bailey A. J. The effect of UV radiation on the structure and properties of collagen. *Polimery* **46**, 379-389 (2001).

[20] Foo, C. W. P., Bini, E., Huang, J., Lee, S. Y., & Kaplan, D. L. Solution behaviour of synthetic silk peptides and modified recombinant silk proteins. *Appl. Phys. A* **82**, 193-203 (2006).

[21] Vassileva, V., Baltova, S., & Handjieva, S. Photochemical behaviour of natural silk-III. Photofading of silk dyed with acid azo dyes. *Polymer Degradation and Stability* **61**, 367-373 (1998).

[22] Baltova, S., Vassileva, V., &Valtcheva, E. Photochemical behaviour of natural silk-I. Kinetic investigation of photoyellowing. *Polymer Degradation and Stability* **60**, 53-60 (1998).
